# Microbial Biofilms: Features of Formation and Potential for Use in Bioelectrochemical Devices

**DOI:** 10.3390/bios14060302

**Published:** 2024-06-08

**Authors:** Roman Perchikov, Maxim Cheliukanov, Yulia Plekhanova, Sergei Tarasov, Anna Kharkova, Denis Butusov, Vyacheslav Arlyapov, Hideaki Nakamura, Anatoly Reshetilov

**Affiliations:** 1 Federal State Budgetary Educational Institution of Higher Education, Tula State University, Tula 300012, Russia; perchikov_roma@mail.ru (R.P.); maxim.cheliukanov@gmail.com (M.C.); anyuta_zaytseva@mail.ru (A.K.); v.a.arlyapov@gmail.com (V.A.); 2 Federal Research Center (Pushchino Scientific Center for Biological Research of the Russian Academy of Sciences), G.K. Skryabin Institute of Biochemistry and Physiology of Microorganisms, Russian Academy of Sciences, Pushchino 142290, Russia; yu_plekhanova@pbcras.ru (Y.P.); setar25@gmail.com (S.T.); 3 Computer-Aided Design Department, Saint Petersburg Electrotechnical University “LETI”, Saint Petersburg 197022, Russia; dnbutusov@etu.ru; 4 Department of Liberal Arts, Tokyo University of Technology, 1404-1 Katakura, Hachioji 192-0982, Tokyo, Japan; nakamurahd@stf.teu.ac.jp

**Keywords:** biofilm, electron transfer, bioelectrochemical systems, microbial biosensor, microbial fuel cells, bioengineering, genetic modification, biochemical oxygen demand, toxicity

## Abstract

Microbial biofilms present one of the most widespread forms of life on Earth. The formation of microbial communities on various surfaces presents a major challenge in a variety of fields, including medicine, the food industry, shipping, etc. At the same time, this process can also be used for the benefit of humans—in bioremediation, wastewater treatment, and various biotechnological processes. The main direction of using electroactive microbial biofilms is their incorporation into the composition of biosensor and biofuel cells This review examines the fundamental knowledge acquired about the structure and formation of biofilms, the properties they have when used in bioelectrochemical devices, and the characteristics of the formation of these structures on different surfaces. Special attention is given to the potential of applying the latest advances in genetic engineering in order to improve the performance of microbial biofilm-based devices and to regulate the processes that take place within them. Finally, we highlight possible ways of dealing with the drawbacks of using biofilms in the creation of highly efficient biosensors and biofuel cells.

## 1. Introduction

**Figure 1:**
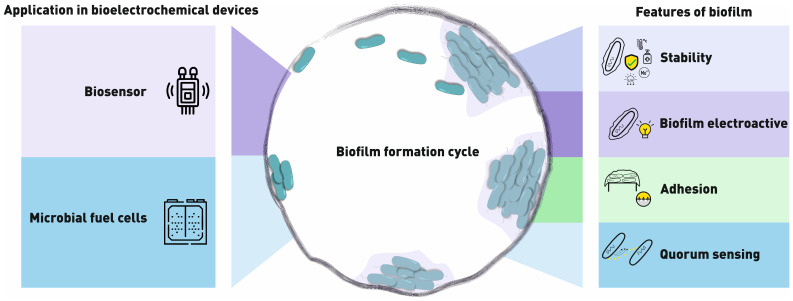
The main characteristics of biofilms for use in bioelectrochemical devices.

Microorganisms can exist in nature in two different forms—free-floating or fixed to any surface as part of a so-called biofilm. The term “biofilm” itself became widespread after a publication by Costerton in 1978 [1]. His theory about the ability of microorganisms to firmly attach to available surfaces became the foundation for the ability to effectively control their properties. This has opened up the possibility of using communities of microorganisms to benefit humans. Important properties of biofilms, such as their resistance to external influences and the ability to firmly adhere to various surfaces without limiting the access of various substances to microorganisms inside the film, make them useful in various fields of industry and medicine. In particular, over the past 10–15 years, there has been an increased interest in using the electrochemical properties of biofilms, especially in the development of biosensors and biofuel cells (Figure 1). Biosensors are analytical devices that consist of a biological recognition element and a transducer. Biofilms can be used in combination with various types of transducers—electrochemical, optical, gravimetric, etc. Such biosensors can be used as tools for evaluating the properties of the biofilm itself, the influence of external conditions on its growth, and the behavior of microorganisms in biofilms. Biofilms can be not only the object of research, but also become the basis of sensor devices for determining various compounds. The principle of operation of such devices consists in changing the properties of biofilms (or in their release of a substance) when a certain compound appears in the medium. At the same time, this compound can both be processed by biofilm microorganisms and have a negative effect on their metabolism. In addition, biofilm-based biosensors can be used to detect not only individual compounds, but their sum. For example, such devices have found wide application in the field of determining biochemical oxygen demand (BOD) or the general toxicity of water [2,3].

The broad substrate specificity of biofilms allows them to be used in another type of bioelectrochemical device—microbial fuel cells (MFCs) designed to generate electricity from organic waste. Typically, MFCs consist of a negatively charged anode and a positively charged cathode, which are placed in separate chambers of the device and separated by a proton-permeable membrane. It is worth mentioning that while any type of biofilm can be used in biosensors, often only electroactive ones, i.e., biofilms formed by electroactive microorganisms embedded in conductive polymers, are used as a part of a biofuel cell system.

**Figure 2:**
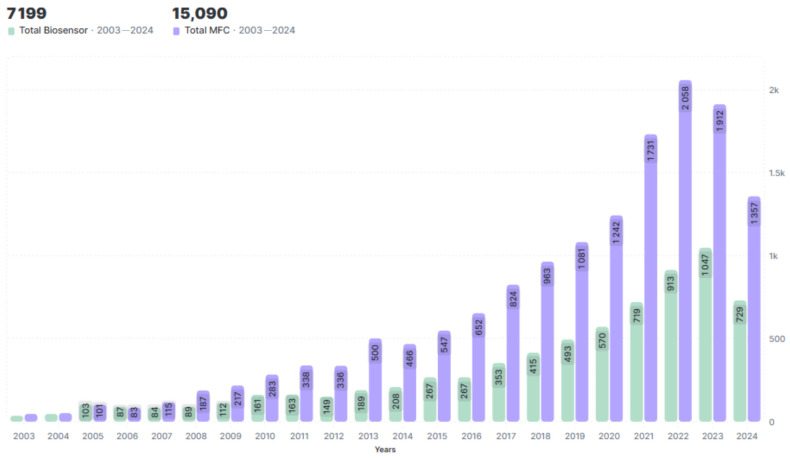
Number of publications in the ScienceDirect database per year with biofilms in bioelectrochemical devices as keywords as of May 2024.

Interest in such devices has been steadily growing over the past 20 years (Figure 2). Research in this field is aimed not only at studying biofilms themselves, but also at discovering new materials to enhance their adhesion, create a microenvironment, and protect them from negative factors. The discovery of novel phenomena and processes that occur within biofilms, such as quorum sensing and the ability to transfer electrons within a population or to external electron acceptors, has led to the development of more efficient bioelectrochemical devices. During this time, several proposed laboratory models have found practical applications and have been commercialized.

This review analyzes the most significant areas of research over the past 10 years. It examines the features of using biofilms in biosensors and biofuel cells and discusses the prospects and potential applications of these technologies. Three databases (Web of Science, SCOPUS, and Google scholar) and two reference managers (Mendeley and Zotero) served as sources of information for searching the necessary data for our paper. In addition, a website search (Google and Yandex) with search operators for publication dates was used to identify potentially relevant information. For all bibliographic sources, keyword searches included the following: biofilms, microbial cells, biosensors, microbial fuel cell, electrochemical biosensors, biosensor design, microbial fuel cell design, electron transport, biorecognition, transduction, biotechnology, bioelectrochemical systems, bioengineering, genetic modification, biochemical oxygen demand, toxicity, quorum sensing, and artificial intelligence.

## 2. Structure and Features of Biofilm Formation

A biofilm is a community of microorganisms attached to a surface with specific polymeric substances that they release into the environment. Up to 80% of all bacteria exist mainly in the form of biofilms [4]. The natural habitat of biofilms is in aquatic and soil environments, such as rivers, lakes, streams, rhizospheres, cave walls, the surface of mineral deposits, and areas around hot springs. Biofilms can also form inside other organisms, including humans—on the surface of the gastrointestinal tract or as part of plaque [4,5,6]. Biofilms form on industrial production lines, heat exchangers, and work surfaces. This leads to corrosion, damage to mechanisms, and contamination of raw materials and food [7]. For the food industry, biofilm contamination can lead to more serious consequences [8], contributing to outbreaks of infectious diseases.

Bacteria that form such surface fouling may acquire new properties during the process. For example, they may develop resistance to antimicrobial agents [9]. Biofilm formation is a microbial defense mechanism that ensures the survival of bacteria. Depending on the place of attachment, microorganisms face various stress factors, such as changes in access to oxygen and nutrients. In order to resist these factors, bacteria modulate their genetic composition [10].

There is no single type of biofilm. The formation of biofilms depends on the type of microbes that form them, their interaction with the host’s immune effectors, as well as the physicochemical and mechanical properties of the microenvironment. Biofilms are diverse in their microbial composition. They can be formed by individual microorganisms (mono-species biofilms) [11,12], by a combination of two or more microbes from the same or different species and strains (multi-species biofilms) [11,13], or even between microorganisms from different taxonomic levels (inter-family biofilms) [14,15].

**Figure 3:**
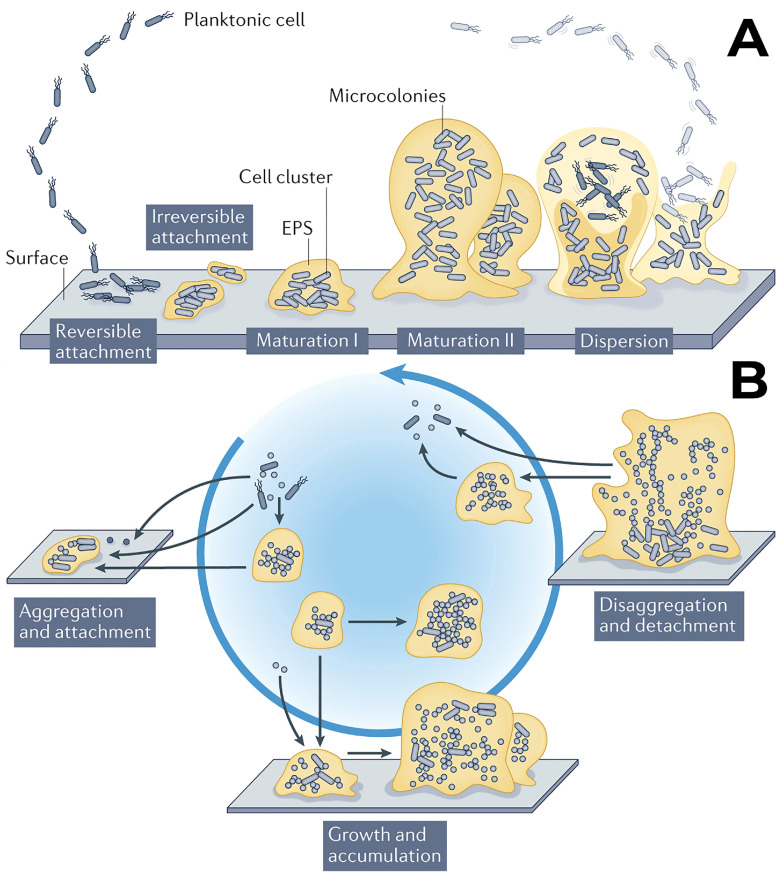
Stages of biofilm development. Reprinted with permission from ref. [16] Copyright © 2022, Springer Nature Limited. (**A**)—The traditional five-stage model of biofilm development. (**B**)—An expanded modern model of biofilm formation.

Despite the high variability of biofilms, there are processes and mechanisms of their development that are common to different species. Traditionally, the biofilm formation model includes five stages: (1) adsorption, (2) adhesion, (3) formation of microcolonies, (4) maturation, and (5) dispersion of the biofilm (Figure 3A).

According to modern concepts, biofilm formation is more of a cyclic process (Figure 3B). During adsorption, bacteria attach to a surface through Lifshitz–van der Waals forces, electrostatic interactions, and acid–base interactions [17]. Most bacteria usually have extracellular appendages of various sizes, structures, and functions. Flagella and pili play a key role in the initial stage of interaction between microorganisms and the surface. Flagella are structures that ensure the movement of bacteria towards a nutrient gradient. Bacteria can attach to other objects using flagella, which is caused by their hydrophobic nature and some of the flagellar motors. Flagella can be either long spiral filaments located outside the cell, such as in *Pseudomonas aeruginosa* [18], or they can be located in the periplasmic space, like in spirochetes [19]. Pili are hair-like structures that surround the body of a bacterial cell. They are protein polymers made up of “pilin” subunits, which are also involved in the movement of both Gram-negative and Gram-positive bacteria [20]. Pili and flagella play an important role in the transition between temporary and irreversible attachment of microorganisms. Certain components of the flagellum are used by bacteria to enhance adhesion to a surface [21]. Some bacterial species (for example, the genus *Vibrio*) switch from using a single polar flagellum during biofilm formation and express a large number of lateral flagella [22].

Once strong bacterial adhesion has been achieved, microorganisms grow and divide by binary fission, for example, during the formation of *Staphylococcus aureus* biofilm on areas of injured bone [23] or asymmetric fission, such as during the formation *P. aeruginosa* biofilms on the surface poly(ethyleneglycoldicyclopentenylethyracrylate) [24]. When forming a “monolayer” biofilm, one of the following mechanisms or a combination of them may be observed:

- Biofilm growth is caused by the expression of agglutins (glycoproteins that coat the bacterial cell wall), such as in the case of *Candida albicans* biofilm formation [25] or expression of fimbriae. Unlike flagella and pili, there are approximately 1000 fimbriae per bacterial cell, such as with *Klebsiella pneumoniae* [26].
- A bacterial cell formed as a result of division separates from the surface and can either attach to a newly formed bio-layer or initiate colonization of other areas of the surface [27].

The aggregation of cells through any of the above mechanisms leads to the formation of microcolonies. As these microcolonies develop, the initial layer of attached cells turns into a multilayered structure. In order to achieve a three-dimensional spatial distribution, bacteria must chemically interact with each other and express certain genes that are responsible for the secretion of an extracellular polymeric substance (EPS). This EPS will serve as a structural support for microcolonies, which then form biofilms. Type IV pili [28] and poly-N-acetylglucosamine [29], produced by some types of microorganisms, play a special role in the formation of microcolonies. These structures ensure the mobility of microcolonies as a cohesive unit, sticking together and forming a biofilm.

After the formation of microcolonies, bacterial aggregation increases, along with the synthesis of the EPS, until optimal cell density is achieved, which is regulated by microbial interactions [30]. At this stage, microcolonies form several layers, which acquire complex structural features as a result of the formation and destruction of biofilms. Bacterial microcolonies form larger aggregates called “macrocolonies” or “towers” [31].

At some point after reaching maturity, the biofilm undergoes partial structural destruction, which can occur as a result of detachment and/or dispersion. The release or loss of a part of the biofilm occurs as a result of mechanical damage, or under the influence of an immune attack by the host. The processes contributing to the destruction of biofilms can be caused by internal changes in the microenvironment: concentrations of nitric oxide, oxygen, temperature, as well as the availability of nutrients [32]. This leads to the depletion of microorganisms in the deepest parts of the biofilm due to stress caused by starvation, hypoxia, and low growth rates. The biofilm begins to activate regulatory mechanisms that contribute to changing the structure of the biofilm. For example, it produces and releases enzymes that destroy the components of the EPS matrix [33], because of the violation of non-covalent interactions with surfactants, such as rhamnolipids [34] and phenol-soluble modulins [35]. These processes lead to the formation of cavities inside the biofilm, which are used by motile bacteria to exit the biofilm. Biofilm dispersion is a well-regulated process. In addition, cells dispersed from the biofilm are more resistant to stress factors than the progenitor cells of the biofilm because during the development of the biofilm, they can change their phenotype.

**Figure 4:**
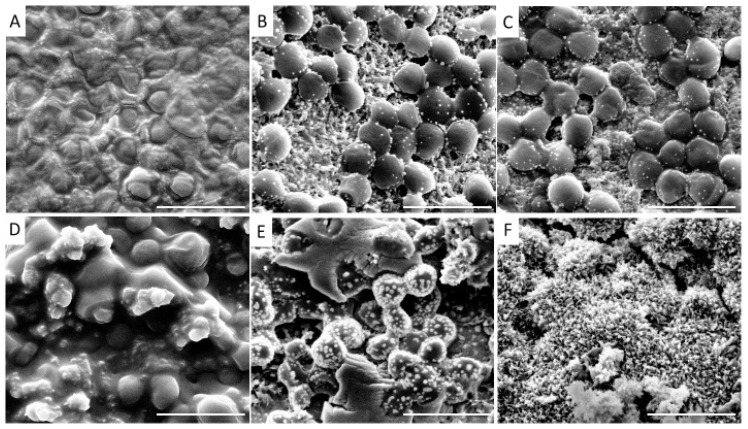
Biofilm of *S. aureus*, visualized using scanning electron microscopy. (**A**,**D**): on silicone; (**B**,**E**): on platinum; and (**C**,**F**): on titanium. The biofilm was grown on a medium without the addition of an antimicrobial component (**A**–**C**) and with the addition of (**D**–**F**). The length of the line corresponds to 2 µm. Reprinted with permission from ref. [36] © 2020 Kirchhoff et al.

It is important to note that the density and, in some cases, the shape of biofilms may depend on the surface they are grown on. Thus, in [36], properties of biofilms formed by *P. aeruginosa* (ATCC9027), *S. aureus* (ATCC6538), *Staphylococcus epidermidis* (ATCC12228), and *Streptococcus pyogenes* (ATCC19615) on medical implants made from three types of material, silicone, platinum, and titanium, was studied. The results of analyzing the biofilms produced based on *S. aureus* are presented in Figure 4.

The material used to grow biofilms contributes to a wide variety of biofilm structures. The densest biofilm was grown on silicone (Figure 4A) and, when grown on titanium surfaces with the addition of antimicrobial materials, the biofilm could not be detected (Figure 4F). The authors also noted the formation of pale structures on the surface of the biofilm (Figure 4B,C,E,F). When an antimicrobial component was added, the density of the biofilm decreased. A similar study on *P. aeruginosa* showed less pronounced changes in morphology [36].

Most biofilms found in nature are multi-species, where each microorganism affects the morphology and architecture, which differ from monospecific biofilms. When using medical devices or in the food industry, such multi-species biofilms represent systems more resistant to external stress factors due to the synergistic effect. Most hospital-acquired infections are caused by *Staphylococcus epidermidis* and *Staphylococcus aureus*, and also *Escherichia coli*, *Klebsiella pneumoniae*, *Acinetobacter baumannii*, and *Pseudomonas aeruginosa* [37]. Various types of medical equipment are subject to fouling by certain types of biofilms. According to a study [38] of the composition of biofilms on the surface of tracheostomy tubes, the most common strains are *Acinetobacter baumannii* (45%) and *Klebsiella pneumoniae* (28.5%). The most dangerous in clinical practice with endotracheal tubes is the presence of bacteria strains of *Pseudomonas*, *Candida*, and *Staphylococcus* [39]. It should be noted that the physiology and metabolic activity of microorganisms within mixed biofilms can be quite difficult to study. The potential dangers of mixed biofilm formation are mainly assessed in the context of medical research, and are associated with methods for antimicrobial surface treatment and the development of antimicrobial drugs.

**Figure 5:**
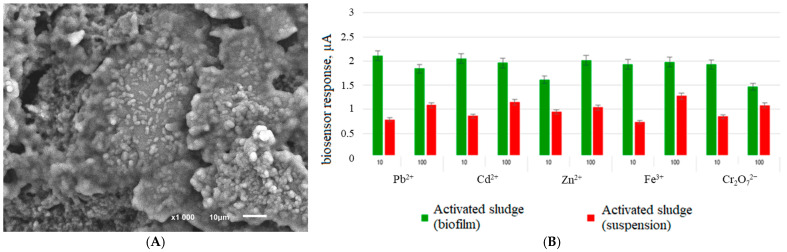
Activated sludge biofilm used to assess biochemical oxygen demand. Reprinted from ref. [40]. Copyright© 2022 Creative Commons Attribution 4.0 License. (**A**) SEM image of the biofilm on the surface of the graphite-paste electrode modified with carbon nanotubes; (**B**) dependence of the response of a biosensor based on a suspension of microorganisms and an activated sludge biofilm on the presence of heavy metal ions.

The resistance of biofilms to various stress factors can be used in a positive way to form biosensor systems in which the analytical signal driven by the metabolic activity of the biomaterial is strongly dependent on the presence of inhibitory substances in the sample. From this point of view, the use of biofilms is advisable for assessing the biochemical oxygen demand [40], and for the formation of biofuel [41]. Figure 5 shows a biofilm of activated sludge (Figure 5A) used to create a rapid assessment device for biochemical oxygen demand.

The use of activated sludge biofilms has made it possible to reduce the negative effects of copper, cadmium, zinc, iron, and dichromate anions on the metabolic activity of the bioreceptor by two or more times (Figure 5B).

The thickness of a microbial biofilm varies depending on the nature of the microorganism, the nutrient substrate, the time of maturation, and the conditions in the microenvironment. For example, the average thickness of monospecific biofilms of *P. aeruginosa* in vitro and *Klepsiella pneumoniae* was 29 and 100 µm, respectively, while a multi-species biofilm consisting of both microorganisms reached an average thickness of 400 µm [42]. In most cases, a mature biofilm is characterized by fixed microcolonies enclosed in an EPS matrix. In addition, the thickness of the biofilm affects its microbial diversity and activity, which in turn affects the effectiveness of substrate transformation processes. For example, in [43] it was shown that thin biofilms are extremely effective in nitrification processes, and thicker films showed less efficient nitrification but improved micropollutant degradation.

Thus, the morphology and physiology of a biofilm depend on the microbial composition, microenvironment, and interactions occurring (or not) between microorganisms and the potential surface on which this biofilm forms. Therefore, depending on the composition and structure of biofilms, they can acquire properties that are useful for their application in bioelectrochemical devices, such as biosensors and biofuel cells.

## 3. Features of Biofilm Formation on Surfaces with Different Architectures and Functionalization

The growth of microorganisms and the formation of biofilms will depend on the type of surface to which microorganisms are attached. In particular, this issue is addressed in detail in the work [44]. When creating biosensor and MFC prototypes, first and foremost, questions arise regarding the material for the working electrode. This material must be non-toxic and preferably highly conductive, and resistant to various external influences. At the same time, such parameters as surface wettability, roughness, stiffness, and surface topography are also important for the growth of biofilms. Therefore, the creation of complex devices such as MFCs and biosensors requires the participation of specialists from various fields—microbiologists selecting suitable strains to create biofilms and biotechnologists picking optimal components to modify the surface of electrodes. By choosing the right surface on which the microbial biofilm will be formed, it is possible to achieve an increase in the lifetime of the working electrode, better performance, and lower maintenance costs. This chapter discusses the specifics of biofilm formation on various types of surfaces.

### 3.1. Formation of Biofilms on the Surface of Plastics and Metals

Most household, medical, and industrial products are created using plastics. The formation of biofilms on polymer materials can have both a positive and negative effect for the operation of products. For example, biofilm formation is usually undesirable in medical institutions. Therefore, fundamental knowledge about the interaction between biofilms and plastics is necessary to create biofilm-resistant materials for medical use [45,46]. In addition, this knowledge can be used in the design of antifouling surfaces for the marine industry [47]. Also, the interaction of plastics with biofilms is important when creating biosensors and biofuel cells, since part of their design involves plastics—support material for electrodes or walls of measuring cuvettes. The negative impact of biofilms associated with plastics may also be due to the accumulation of pathogens and the transfer of genes for resistance to antibiotics, metals, etc. [48,49].

**Figure 6:**
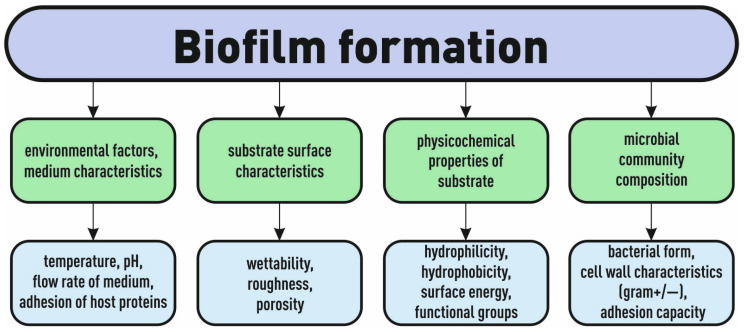
Factors influencing biofilm formation.

In [45], the surface properties of materials made from non-biodegradable polyethylene terephthalate (PET) and a biodegradable polymer based on polylactic acid (PLA), as well as their attachment to microorganisms in their natural habitat (seawater), were studied. Changes in surface roughness were monitored after 24 h using atomic force microscopy (AFM) and SEM. With the help of SEM, an accumulation of microorganisms was detected on two plates. The results showed that an increase in surface roughness as a result of conditioning led to the rapid attachment of microorganisms. The authors emphasize that one of the most important factors for the successful adhesion of microorganisms is surface conditioning, determined by a variety of factors such as environmental characteristics, surface properties, material properties, and microbial composition (Figure 6).

In [50], the development of biofilms on various types of plastic was monitored under standard conditions and in low-light conditions. The authors used low- and high-density polyethylene (LDPE and HDPE, respectively), polypropylene (PP), and polyvinyl chloride with two typical additives (PVC DEHP and PVC DINP), and glass as an inert control. After one week of incubation, the PVC surface showed a more developed biofilm both when exposed to the environment and in dim light compared to other plastics and slides, with a large area of the surface covered with a biofilm matrix and individual cells. After two months of incubation, all plastic and glass surfaces were covered with biofilm. The authors conclude that the differences in the composition of bacterial communities associated with various plastic surfaces appear to be greater at the initial than at later stages of biofilm formation. In the later stages of biofilm formation, these differences were more pronounced when the samples were stored in dim light than when the samples were well illuminated. In sum, this study reveals various schemes of colonization by the microbial community, depending on the properties of the plastic and the effects of solar radiation.

The positive effects of biofilm formation on plastics include their impact on the environment—for example, on the regulation of desorption of heavy metals from plastics through the formation of metal complexes [51,52]. For example, it has been shown that copper is more difficult to desorb from biofilm-coated polyethylene than from polyethylene without a microbial community [53]. The extracellular polymer matrix of phototrophic biofilms may exhibit high affinity for metal cations [54]. Such biofilms coated with metal cations can be used in bioelectrochemical devices.

**Figure 7:**
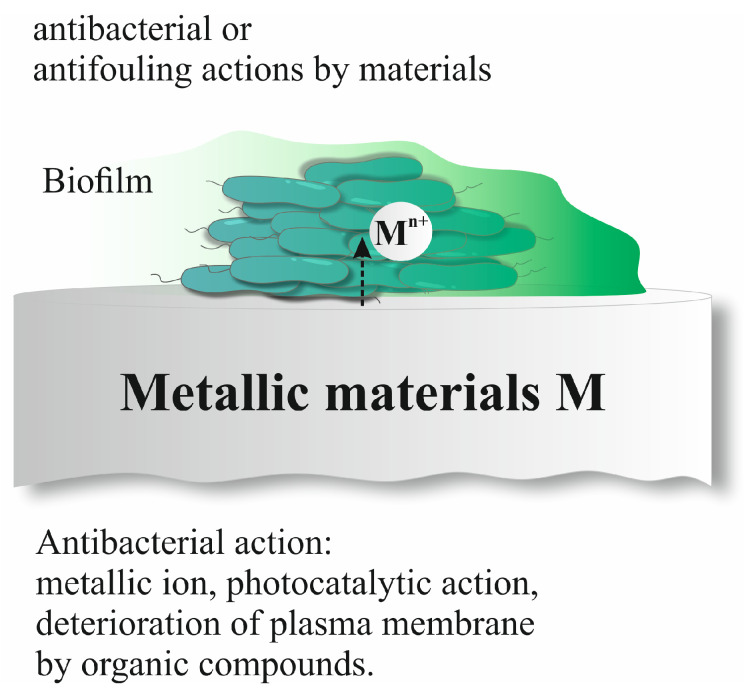
Growth of biofilms on metals, adapted from [55].

As for the growth of biofilms on metal surfaces, metal ions dissociated from metals in an aqueous medium play an important role, as shown in Figure 7. Metal materials usually have a negative static charge, as do the surfaces of bacterial cells. It may be necessary for bacteria to eliminate the effect of electrostatic repulsive forces in order to attach to the metal surface.

Biofilms contain a variety of polymers consisting of proteins, polysaccharides, lipids, and nucleic acids that help them attach to surfaces. Many metal ions can embed themselves between these polymers or bind to proteins in the form of a chelate, contributing to an even stronger attachment of biofilms [56]. Metal ions can also form complexes with proteins that are part of bacterial membranes, which initially act as receptors or carriers [57]. *Shewanella* and other genera of bacteria that grow on metal surfaces can use metals such as iron and magnesium as terminal electron acceptors during anaerobic respiration [58]. Heavy metals bind to the cell wall via inner sphere complexation (e.g., no interlayer water molecules) with multiple anionic oxygen ligands [59].

Metals such as iron, calcium, copper, zinc, manganese, and chromium can participate in the complexation with bacterial proteins. Various metals are introduced into bacterial cells through metal-binding transporters. As a rule, divalent metal ions have their own carriers on bacterial membranes. These mechanisms contribute to the realization of strong interactions between metallic materials and bacteria, with the formation of biofilms and overcoming various repulsive factors [55].

### 3.2. The Effect of Surface Functionalization on Biofilm Formation

Bacterial adsorption is associated with such properties of the cell surface as bacterial hydrophobicity, surface charge, cell size, and also the properties of the environment [60]. Stable bacterial adsorption can be achieved through short-range forces such as orientation forces, hydrogen bonds, hydrophobic interactions, and ionic or covalent bonds [61,62]. Moreover, their adsorption based on physico-chemical reactions is successfully used for the vast majority of bacteria that attach to natural surfaces [63].

The unevenness or porosity of the surface plays an important role in the fixation of bacteria. In addition, the presence of functional groups on the surface of materials that come into direct contact with the outer membrane of bacteria and EPS has a great influence [64]. These groups are also involved in physicochemical reactions at the interface. Therefore, the functionalization of surfaces can affect subsequent bacterial adhesion. In addition, according to the theory of the double electric layer, electrodes modified by functional groups in a bioelectrochemical cell can also change the parameters of the double electric layer around the electrode itself, and then create a suitable electrical microenvironment for exoelectric processes. Interestingly, bacterial attachment helped to stabilize HSO_4_^−^ modified polyaniline and avoid HSO_4_^−^ detachment, which showed a mutually beneficial relationship between HSO_4_^−^ groups and attached microbes [65]. In another case, based on the molecular affinity between lectin (naturally present in type I bacterial pili) and mannose, mannose was chosen to modify the colonized surface and accelerate the attachment of bacterial strains with type I pili and the appearance of electron transfer [66]. More specifically, modification by functional groups enhances bacterial adhesion due to the effects of: chemical bonding, electrostatic interaction, hydrophobization/hydrophilization of the surface, and surface roughness.

Surface functional groups have a significant effect not only on adhesion, but also on the growth and structure of the biofilm, since they are directly involved in physico-chemical interactions at the interface [67]. It was found that the presence of -SH and -NH groups from gamma-aminopropyltriethoxysilane in polyvinyl alcohol increases the protection of microorganisms from aggressive environmental conditions. For example, the researchers managed to grow a dense biofilm resistant to a heavy metal—Cr(VI) [68]. These groups were additional reaction sites for chromium deposition. It is important to note that modification of the electrode surface should increase biocompatibility for bioelectrochemical devices. Thus, in [69] bamboo carbon tubes were modified with C=O and C-H groups, which led to a 63% increase in colonies of forming units (CFUs) on their surface than on the surface of a graphite tube anode.

The materials used as anode electrodes must have several specific characteristics to improve the interaction between the electroactive biofilm and the surface of the material. The most important characteristics are: (1) good scalability; (2) corrosion resistance; (3) high conductivity; (4) developed surface area; (5) biocompatibility; (6) environmental friendliness; and (7) low cost [70].

**Figure 8:**
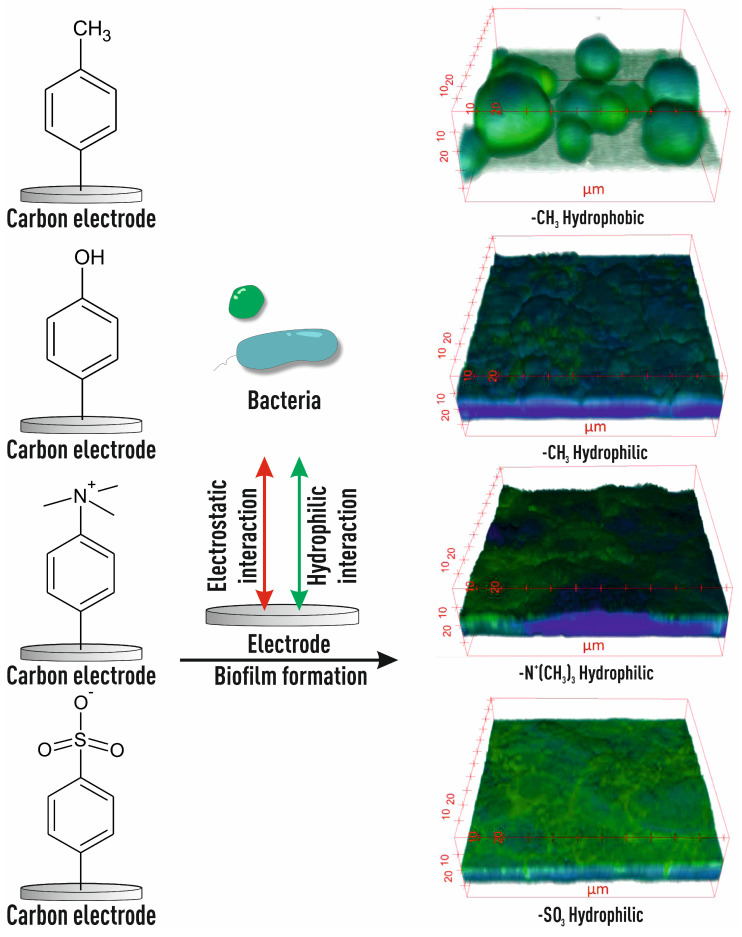
Confocal laser scanning microscopy three-dimensional images of biofilms on glass carbon modified by various functional groups. Adapted with permission from ref. [72] © 2013, American Chemical Society.

It has been proved that changing surface potential, wettability, and other related physicochemical properties by modifying functional groups affects the morphology and structure of the biofilm. The hydrophobicity of the surface has a significant effect on the thickness of the biofilm, which is associated with poor diffusion of nutrients near the hydrophobic surface [71]. In [72], surfaces with controlled hydrophobic and hydrophilic properties were studied on surfaces made of glass carbon modified with -OH groups, -SO_3_^−^ and -N^+^(CH_3_)_3_ by electrochemical reduction of the corresponding aryldiazonium. The maximum thickness of the biofilm increased in the order -N^+^(CH_3_)_3_ > -OH- > -SO_3_^−^. In addition, modification of functional groups also leads to a change in the structure of the biofilm community, which is consistent with the generally recognized fact that positively charged surfaces are more preferable for electroactive microbes (for example, *Geobacter*), and as a result, biofilms with increased electrical activity are formed (Figure 8).

It can be seen from the presented microphotographs that a more uniformly distributed biofilm with a larger coating area, biomass, and *Geobacter* ratio is formed on the surfaces of electrodes modified with hydrophilic or positively charged functional groups than on surfaces modified with hydrophobic, neutral, or negatively charged groups.

Modification of the electrode surface with organosilicates improves the physical, chemical, and mechanical properties of the surfaces, and most importantly enhances microbial adhesion. Factors influencing the nature of the modification of the organosilane surface include the concentration of surface hydroxyl groups, the type of surface hydroxyl groups, the hydrolytic stability of the bond formed, and the physical characteristics of the material [73]. The chemical structure of silane can be modified to achieve the required characteristics, such as a given hydrophobicity, surface charge, certain functional groups, or acid–base properties necessary to improve the formation of a useful biofilm [74].

Pretreatment of the surface with partially or completely ionized gas (plasma) is an ideal strategy to improve the interaction of bacteria with electrodes and the efficiency of the electric current. Such a process modifies the surface of metallic materials through chemical or physical processes at the atomic or molecular level [75]. Typically, the plasma-forming gases used for this process are argon, nitrogen, oxygen, carbon dioxide, and ammonia. Plasma treatment enhances the initial adhesion of microbial cells, which in turn increases biofilm formation [76]. The wettability of the surface determines the adhesive properties of microbial cells. The formation of functional groups by plasma promotes wettability, which leads to an increase in adhesive properties and an increase in surface energy [77].

**Figure 9:**
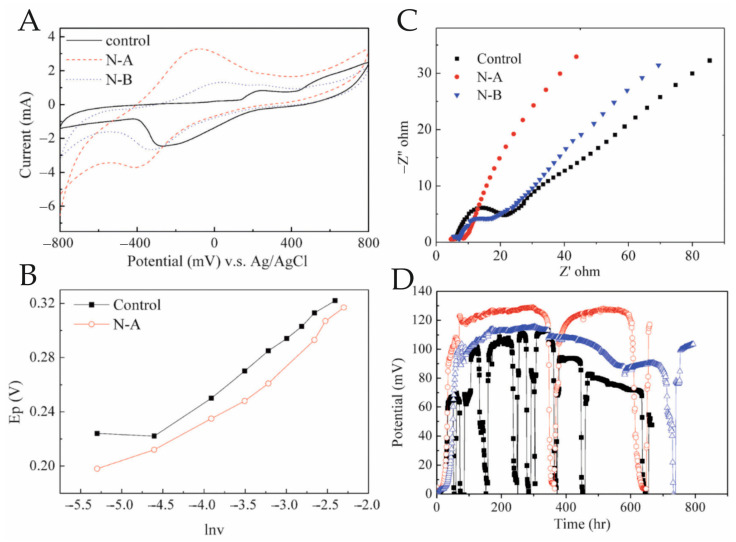
(**A**) Cyclic voltammograms of various electrodes in MFC inoculated with anaerobic sludge, and (**B**) graph of the dependence of Ep on lnv, scanning speed: 5–100 mV s^−1^. (**C**) Nyquist graphs for carbon paper with and without N^+^ ion in a phosphate buffer. (0.67 M K_2_HPO_4_ and 0.67 M KH_2_PO_4_) with 2.5 мM Fe(CN)_6_^3−^ and 2.5 мM Fe(CN)_6_^4−^. (**D**) Voltage outputs for the developed systems. Reprinted with permission from ref. [79] © Royal Society of Chemistry 2024.

The use of atmospheric and oxygen plasma on various carbon electrodes has been extensively studied. Plasma treatment of electrode surfaces improves electron transfer and increases the generated current. Pretreatment of the electrode with a 25 W radiofrequency oxygen and nitrogen plasma led to an increase in the initial anode current from the inoculum and a higher rate of bacterial adhesion on the electrode surface and provided higher biofilm growth compared with untreated electrodes [78]. Plasma implantation of nitrogen ions was used to modify the anode materials in a microbial fuel cell (Figure 9). A thicker layer of cells was formed on the treated anode with altered surface roughness and hydrophobicity, which, in turn, enhanced biofilm formation and increased electricity generation. The three gradients of dosage were defined as the control, N-A, and N-B, respectively [79].

Perhaps the most common method of modifying electrode surfaces in recent years has been the use of carbon nanomaterials. The surface area and pore volume of carbon nanotubes (CNTs) used to immobilize bacteria can be further increased by modifying the surface or changing the synthesis conditions. Such modifications increase the dispersion of CNTs, causing favorable structural changes that contribute to the formation of biofilms on their surface. Since the hydrophobic nature of primary CNTs limits their practical application, studies have recently been conducted to study the mixing of CNTs with materials such as conductive polymers [80,81], precious metals [82], and chitosan covalently crosslinked with ferrocene [83]. The resulting composites have higher electrical conductivity, better stability, and the ability to work in a wide range of physico-chemical conditions (pH and temperature). Non-toxic CNT nanocomposites (polypyrrole or polyaniline) with high conductivity can be used in MFCs to improve electron transfer from microbes to the anode [84,85]. The resulting polymers are able to reduce the cytotoxicity of CNTs by increasing their solubility, which allows them to be used effectively in bioelectrochemical devices. In some cases, this modification allows for the direct transfer of electrons from the biomaterial to the electrode. Natural biocompatible polymers such as chitosan can also reduce the toxicity of CNT nanoparticles and lead to an increase in the specific power of the electrodes modified by them [86]. CNTs are able to integrate into the polysaccharide matrix of biofilms, forming a conductive network that allows the transfer of electrons directly from redox shuttles located in the EPS [87].

Chemical modification of the surface with redox compounds is also an effective approach to obtaining biofilms with desired properties. Covalently immobilized neutral red (NC) and methylene blue (MB) have high electrochemical activity, increase biofilm adhesion, and contribute to high power generation [87,88]. In [89], the effect of surface hydrophobicity on electron transfer by cytochromes was studied for the first time. C-type cytochromes on a gold electrode modified with hydrophilic groups -COOH and -OH showed a fivefold decrease in electron transfer resistance compared with the modified -COOH electrode, which indicates that the hydrophilic surface stimulates the activity of c-type cytochromes.

## 4. Modification of Biofilms to Increase the Efficiency of Bioelectrochemical Devices

To effectively use microbial biofilms as part of bioelectrochemical devices, it is necessary to ensure the transfer of electrons from the active centers of microbial enzymes to the electrode surface. With the development of research on electroactive biofilms, their modification has become a promising strategy for the development and improvement of bioelectrochemical systems for various applications in the fields of biosensor analysis, green energy, etc.

Electron transfer can be carried out by outer membrane proteins or enzymes, as well as other conductive structures. The mechanisms of biofilm electroactivity are divided into direct and indirect electron transfer. The efficiency of direct electron transfer depends on the architecture of the biofilm on the anode surface. Cytochromes and pili, or nanowires, can form a dense network in the biofilm matrix. Conductive pili are responsible for the transfer of electrons between the layers of biofilms. In addition to direct electron transfer, there is an indirect transfer mechanism carried out by mediators. The most well-known electronic shuttles secreted by electrogenic bacteria are phenazine derivatives and flavins produced by *Shewanella* [90].

In the next section, the modification, adjustment, and influence of these parameters on the growth and electroactivity control mechanisms of electroactive biofilms (EABs) in their various applications will be discussed.

### 4.1. Genetic Modification of the Direct Transfer Pathway in Electroactive Biofilms

In the case of direct electron transfer, genetic engineering tools have recently been actively used to modify the mechanisms of electroactivity. Researchers mainly use the activity of genes responsible for the production of cytochromes and pili. This allows them not only to improve the performance of electroactive cells, but also to influence the morphology of the biofilm itself.

Strains of *Geobacter sulfurreducens*, deprived of four of the five cytochrome complexes of the outer membrane (extABCD+ strain), grow faster and produce a higher current density than the wild type grown under identical conditions. The removal of these complexes leads not only to the formation of denser biofilms, but also to an increase in the electron transfer rate compared to the wild strain [91]. A similar effect is produced by modified *Shewanella oneidensis* MR-1, capable of expressing OmcA, which was further used to create a mixed bacterial MFC with higher electrochemical characteristics. When modified bacteria are added to the system, a more compact biofilm with increased electrochemical characteristics is formed [92].

**Figure 10:**
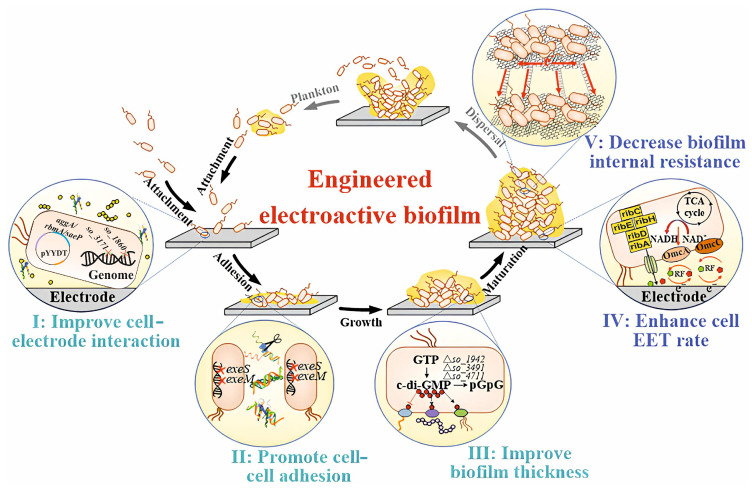
Scheme of modular technology for creating full-cycle biofilms. Reprinted with permission from ref. [93] © 2023 Feng Li et al. Using the methods of synthetic biology modular strategy via regulating the full-cycle biofilm formation process, a highly conductive biofilm based on the *S. oneidensis* model electrogen was obtained. To increase the ability to form biofilms, the authors increased the cell coverage area on the electrode surface by engineering the initial contact stage (I), promoted cell adhesion at the adhesion stage (II), and increased the vertical expansion of the biofilm to enhance the formation of a 3-dimensional (3D) structure at the biofilm growth stage (III). To increase the conductivity of the biofilm, the synthesis of c-type cytochromes of the outer membrane (cuts) and riboflavin was enhanced in order to increase the rate of extracellular electron transfer of each cell of the natural electroactive biofilm at the stage of stable maturity (IV). Then, a 3D, self-assembled artificial electroactive biofilm was created in the mature dispersion stage (V).

An artificial electroactive biofilm of *Shewanella oneidensis* with high electrical conductivity is presented in [93]. Figure 10 shows a diagram of a modular technology for creating full-cycle biofilms.

In the process, the synthesis of outer-membrane c-type cytochromes and riboflavin were activated, which allowed the increase in the rate of biofilm formation at the stage of stable maturity. Based on this, a self-assembling artificial biofilm with increased electron capacity and reduced internal resistance was additionally constructed, which improved power density indicators.

*Cupriavidus metallidurans* CH34 pJBpleD* has constitutively active diguanylate cyclase (DGC), which increases c-di-GMP levels. It is noteworthy that DGC expression in the *C. metallidurans* strain CH34 pJB*pleD* led to higher biofilm formation and increased electric current generation by up to 560%. In addition, *C. metallidurans* CH34 pJBpleD* showed elevated levels of transcripts associated with c-type cytochromes. Scanning electron microscopy revealed a dense extracellular matrix with an increased content of exopolymer substances in the biofilm on the electrode surface. The results of this study suggest that higher levels of c-di-GMP activated the formation of an electroactive biofilm on the electrode, enhancing its exoelectrogenic activity [94].

The work described above touched upon modifications related to the operation of cytochromes; however, as mentioned earlier, it is possible to improve the operation of pili too. In the study [95], seven genes associated with the extracellular synthesis of polysaccharides and proteins were studied to stimulate the synthesis of the EPS matrix. It was found that the expression of the *mxdB* gene, encoding glycosyltransferase from *S. oneidensis,* and *pilA*, encoding the type IV pili assembly protein from *P. aeruginosa*, led to significant extracellular synthesis of polysaccharides and proteins, with the help of which the genetically engineered strain EnBF2 was obtained, demonstrating a higher rate of biofilm formation and the rate of extracellular electron transfer (EET).

Multi-species microbial communities form biofilms with certain spatial patterns. Miaoxiao Wang and colleagues [96] investigated the spatial structure and mixing of a constructed synthetic consortium consisting of two mutualistic strains of *Pseudomonas stutzeri*. It was found that the consortium self-organizes into the so-called spatial pattern of a “bubble explosion” with a low level of mixing. Interestingly, when the genes encoding type IV pili were removed from both strains, the mixing in the spatial pattern increased and the productivity of the entire community increased. Thus, type IV pili play a role in facilitating the spatial mixing of different populations in surface-attached microbial communities, which allows for community-level property management.

**Figure 11:**
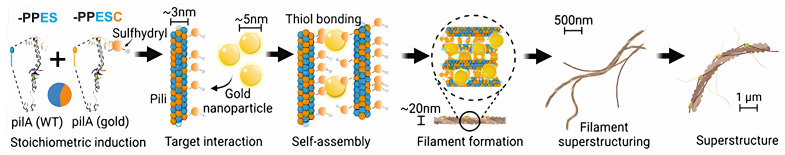
Schematic for co-expression of wild-type + mutant PilA monomers assembling into heterogeneous pili designed for specific target interactions, forming long-range filament bundles of conductive pili culminating in superstructures. Adapted from ref. [97].

The pilin protein can be modified to increase the conductivity of filaments and create biohybrid organometallic structures. Thus, a strain of *Shewanella oneidensis* was developed, which expressed abundant conductive *Geobacter* pili during aerobic cultivation in liquid culture. The pilin expressed by *S. oneidensis* was modified by cysteine to bind to gold. As a result, the pili self-organized into biohybrid filaments in the presence of gold particles. Figure 11 shows a scheme for obtaining these conductive threads [97].

The authors suggested that the addition of a cysteine label opens the way to the creation of biohybrid organometallic structures in which gold nanoparticles serve as connectors or gaps between the saws.

The electroactivity of fields can also be controlled by changing their aromatic composition. For example, in *Cupriavidus necator* H16, various aromatic modifications of type IV pilin proteins were performed to establish structural and functional relationships of conductivity and the effect this has on their structure. First, the authors confirmed that the conductivity of microbial pilus proteins can be changed by a combination of aromatic composition and modification of the secondary structure of the monomer. Secondly, it is demonstrated that the expression of conductive type IV nanowires affects the redox properties of recombinant *C. necator* strains, allowing bacterial cells to electrochemically interact with the environment with more than a ninefold increase in peak oxidative current compared with the wild type. Taken together, these results support the hypothesis that conductive microbial nanowires may consist of PilA proteins [98].

As has been shown, the modification can affect cytochromes in combination with pili. Thus, by genetically fusing a minimal cytochrome domain (MCD) with the curli protein CsgA in *Shewanella oneidensis* MR-1, an electroactive cytochrome-fused curly network was developed. This strain provided a higher output voltage (2.4 times increase) and power density (2 times increase) compared to the wild-type MR-1 when used in MFCs [99].

A mutant strain of *Geobacter sulfurreducens Δgsu1771* was also obtained using the marker-free gene deletion method to evaluate and characterize the role of this transcription regulator in the expression of genes associated with EET. The removal of this gene delayed the growth of microorganisms in the acetate/fumarate medium; however, a more efficient recovery of soluble and insoluble Fe(III) oxides took place. DNA–protein binding assays have shown that the gene GSU1771 directly regulates transcription of *pilA*, *omcE*, *omcS* and *omcZ* genes. In addition, mutant biofilms with *gsu1771* deficiency are thicker than those of wild-type strains [100].

### 4.2. Modification of Indirect Electron Transfer in Electroactive Biofilms

Most microbial-based systems require the addition of artificial mediators for electron transfer, which may limit the applicability of devices based on them. Therefore, the ability of microorganisms to secrete endogenous mediators is in demand when creating bioelectrochemical devices. Since phenazine and flavin derivatives are responsible for indirect electron transfer, emphasis is placed on enhancing the synthesis of these mediators when improving this transfer pathway. This is achieved by introducing special genes or regulators that affect the metabolism of these derivatives.

In [101], the pathway of phenazine biosynthesis from *P. aeruginosa* was introduced to *E. coli*. This biosynthetic pathway contains a phenazine cluster of seven genes, namely *phzABCDEFG* (*phzA-G*), responsible for the synthetic formation of phenazine-1-carboxylic acid (PCA) from chorismic acid, and two additional phenazine auxiliary genes *phzM* and *phzS*, catalyzing the conversion of PCA to pyocyanin (PYO). The engineered *E. coli* cells were used to create a microbial fuel cell with improved characteristics, demonstrating an increase in maximum power density [101]. The electroactivity of microorganisms is controlled by many genes, so manipulations with individual genes have limitations. An exogenous global IrrE regulator was used for *Pseudomonas aeruginosa* P3-A-11. As a result, four mutants with higher electroactivity were obtained, among which the mutant 11/M2-59 not only demonstrated maximum power density, but also showed higher salinity tolerance. Additionally, an increase in the amount of phenazines contributed to an increase in the output electrical power. It is noteworthy that IrrE had a positive effect on electroactivity even without regulators such as PmpR and RpoS [102]. Thus, global regulator technology is an effective approach for simultaneous optimization of electroactivity and resistance to salt stress.

In order to increase flavin biosynthesis and thus improve the electron transfer rate, the *ribADEHC* gene operon was overexpressed in *Shewanella algae*-L3F, which increased the power density of the MFC [103]. Juntao Zhao and colleagues enhanced free and riboflavin-mediated (RF) extracellular transport through various combinations of structural genes (RF operons) and regulatory elements and overexpression of MTRc. They also further improved biofilm formation due to overexpression of the cell division inhibitor *sulA* [104]. In the work, the possibilities of synthetic biology and materials science were combined (multi-walled nanotubes and graphene oxide were used) to increase the efficiency of an electrochemical device.

In addition to gene modification of electron transport pathways and genes responsible for the production of exogenous mediators, an exogenous mediator can also be introduced into devices, which will facilitate indirect transfer. For example, in [105] methylene blue (MB) was used as a transient mediator to enhance EET between the biofilm and the electrode. MB induces a heterogeneous distribution of extracellular polymeric substances, in particular a large number of proteins, which can stimulate multistage electron transfer from granules to electrodes or granules nearby. In addition, the surface charge of the system becomes less negative when MB is added, which reduces the electrostatic repulsive interaction between granules and bacteria. At the same time, the microbial community is enriched with exoelectrogens (i.e., *Geobacter*).

Another way to modify biofilms is the use of carbon dots (CDs). Modification of bacteria with acetogenic CDs doped with nitrogen enhances EET and the formation of an electroactive biofilm, which subsequently improves the production of acetate from CO_2_ in microbial electrosynthesis (MES). The CD modification increased the biofilm thickness by 86%, which may be due to increased secretion of flavins, which enhance interspecific signal transmission. When modified with N-CDs, expression of functional genes encoding cytochromes (*cydB*, *cybH*, and *CcdA*) was significantly enhanced [106].

### 4.3. Other Methods of Forming and Stimulating the Growth of Electroactive Biofilms

In addition to enhancing electroactive properties, it is possible to influence the formation and growth of electroactive biofilms. This includes artificial biofilm formation, stimulation of polysaccharide matrix synthesis, and inhibition of the growth of non-electrogenic organisms in communities.

**Figure 12:**
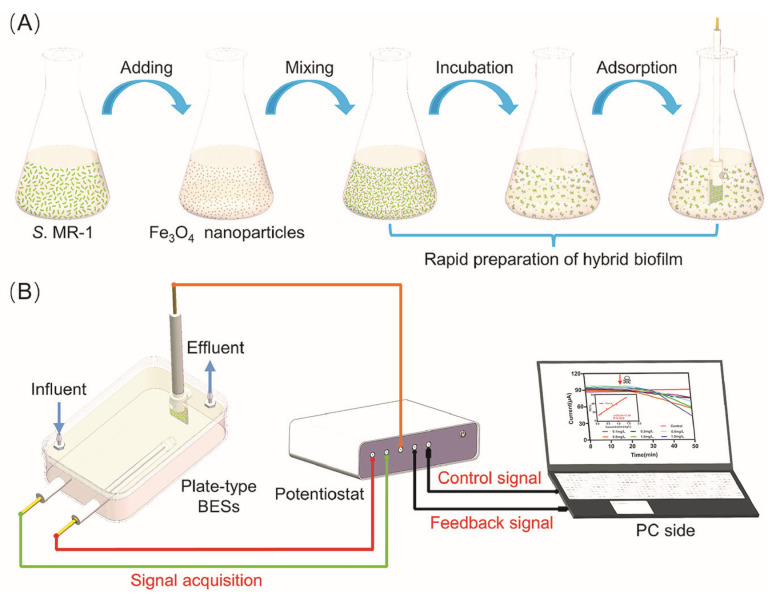
Schematic diagrams of magnetically constructed EAB biofilm preparation (**A**) and instant water toxicity detection (**B**). Reprinted with permission from ref. [107] © 2023 Elsevier B.V. All rights reserved.

As for the artificial formation of biofilms, it can be noted the development of a new method for the rapid creation of an EAB of *S. oneidensis* MR-1 using magnetic adsorption. The formation scheme is shown in Figure 12.

Magnetic Fe_3_O_4_ nanoparticles mixed with a suspension of microorganisms were used to produce EABs. Using a carbon cloth and a magnet, a magnetic electrode was formed, which was immersed in a suspension of microorganisms and a magnetically constructed EAB biofilm was obtained. The results demonstrate that the biofilms were magnetically constructed in less than 30 min, and at the same time they generated stable currents even under continuous flow conditions. Biofilms with a magnetic design provided instant detection of water toxicity, and sensitivity increased with decreasing magnetic field strength. The low magnetic field strength led to the formation of a loose biofilm structure, which facilitated the penetration of toxic pollutants [107].

Fengjie Zhao and colleagues developed a lithographic strategy for creating conductive biofilms of *Shewanella oneidensis* by controlling the expression of the CdrAB aggregation protein using a blue-light-induced genetic scheme. This controlled deposition allowed the formation of *S. oneidensis* biofilms on transparent electrode surfaces, and tunable conductivity was demonstrated depending on the size of the pattern [108].

Biofilm formation can also be stimulated with the help of lysozymes, regulating the content of the polysaccharide matrix. It is assumed that lysozymes can improve the membrane permeability of positive bacterial cells and, thus, increase the EPS content in the activated sludge. The characteristics of the electrochemical activity, surface morphology, and community structure of the anode biofilm indicate that an increase in the EPS content promotes the adhesion of mixed bacteria in the activated sludge on the electrode and leads to the formation of denser biofilms with better conductivity [109].

An interesting approach to stimulating biofilm growth is to influence the mechanisms of quorum sensing (QS). For example, N-butyryl-l-homoserine lactone (C4-HSL) was added as a typical QS signaling molecule to the biocathode in an MFC. The results showed that the efficiency of sulfate reduction and the stability of the biocathode were higher with the regulation of C4-HSL [110]. It has also been demonstrated that it is possible to enhance the sensing of quorum by using acylase in an anode biofilm to improve the determination of naphthenic acid concentrations in a biosensor based on a microbial electrochemical cell. The addition of acylase increased the relative expression of QS-associated genes (*las*R, *las*I, *rhl*R, *rhl*I, *las*A, and *Lux*R) by 7–100%, along with an increase in the number of known electroactive bacterial genera such as *Geobacter* (from 42% to 47%) and *Desulfovibrio* (from 6% to 11%) [111].

Zhiyuan Yang and co-authors have developed a “bidirectional” microbial community regulation method that can selectively inhibit Gram-positive, non-electrogenic bacteria and improves conduction and electron transfer between electrogenic bacteria and an electrode in an MFC using an Au/Nisin nanocomposite [112].

## 5. Biofilms as Recognition Elements of Biosensors

Despite all the benefits of using biofilms (both natural and artificial) as part of biosensors, they have serious disadvantages that limit their applications. These include, first of all, stability, specificity, and sensitivity compared, for example, with purified enzymes. In this regard, biofilms are most widely used as a recognition element of a biosensor in those areas where high selectivity is not mandatory. That is, in an analysis of objects that either shows the total quantity of substances contained in the sample (BOD analysis), or a “Toxicity sensor” for a wide range of substances of a certain class (heavy metals, pesticides, and general toxicity), where the very presence of a contaminant in the sample is important, and not its exact quantitative determination.

In the last decade, early-warning, biofilm-based biosensors have been most widely used in environmental applications and water quality monitoring, where their self-maintenance and regeneration properties can be used most effectively. The main task of these biosensors is to provide timely warning of the presence of composite pollutants with an acceptable low selectivity of the sensor. Accordingly, most of the works published on this topic in the last 10 years are aimed specifically at creating devices that would identify different types of substances that can be found in aquatic environments, and the presence of which must be promptly reported. Such compounds include heavy metals, organic toxicants, nitrates, etc. In addition, the most important properties of biosensors based on microorganisms include the fact that they are able not only to detect pollutants in samples, but also often to simultaneously purify these samples, which makes such devices extremely promising for use. Such devices should rather be classified as microbial fuel cell-based biosensors, since by their structure they are often standard MFCs, which perform an additional function—the quantitative determination of pollutants [113]. Let us take a closer look at each of the classes of compounds that can be determined using microbial biosensors based on biofilms.

### 5.1. BOD Biosensors

One of the areas of application of biofilm-based biosensors is related to environmental monitoring. And the most important indicator of the degree of water pollution is the biochemical oxygen consumption. According to the definition, BOD is the amount of dissolved oxygen consumed over a set time and under certain conditions during the biochemical oxidation of organic substances contained in water. BOD is usually determined after 5 days of incubation (BOD_5_); however, the content of some compounds is more informatively characterized by the value of BOD in 10 days or during the period of complete oxidation (BOD_10_ or BOD_full_, respectively). Biosensors allow rapid analysis of water pollution when results can be obtained within a few minutes. And in this case, the wider the substrate specificity of the biosensor bioreceptor, the more accurately it will show the value of BOD. Biosensors for determining BOD are the most widely utilized microbial biosensors for commercial use and many reviews are devoted to this topic [114,115]. The development of BOD biosensors began around 40 years ago with the first biosensor proposed by the Japanese researcher Isao Karube in 1977. The biosensor was based on *Clostridium butyricum* IF0 3847 bacteria [116]. Then, the artificial microbial biofilm was prepared by spreading a suspension of *C. butyricum* and collagen on a plate, then treating it with a glutaraldehyde solution and drying it. Subsequently, yeast cells with broad substrate specificity were used to determine the BOD index [117]. Through this study, a microbial biofilm was developed in which yeast was sandwiched between two nitrocellulose membranes, and the exchange of the biofilm was facilitated, leading to the practical application of the BOD biosensors. In further studies that were carried out, consortia of microorganisms specially selected [118] or existing in real conditions, for example, consortia of activated sludge [119], were applied.

**Figure 13:**
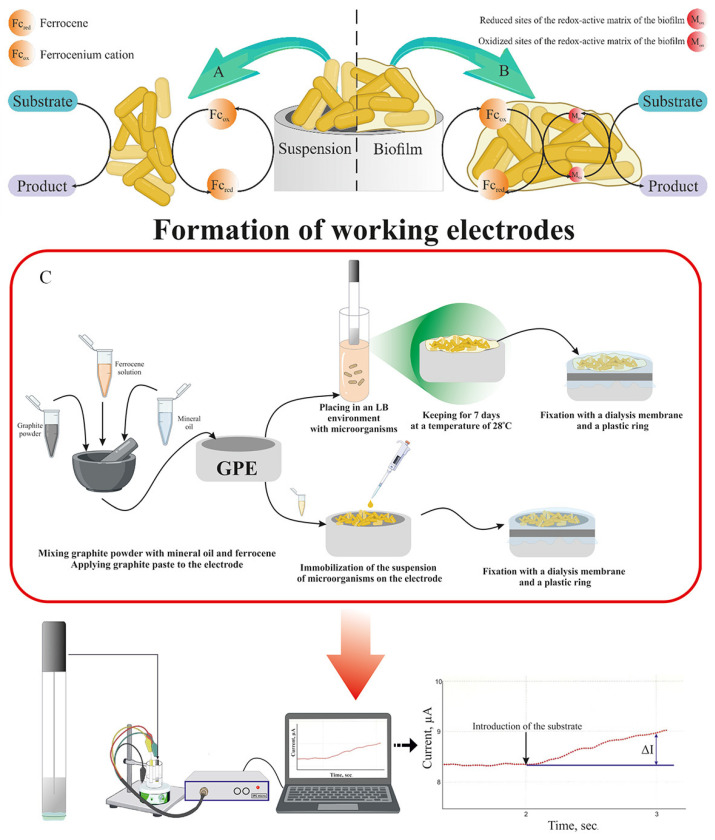
The mechanism of electron transfer in the studied biosensor systems. Reprinted from ref. [125]. Copyright © 2024 Creative Commons Attribution 4.0 License. (**A**)—in the system “graphite paste electrode—ferrocene—immobilized microorganisms”; (**B**)—in the “graphite paste electrode —ferrocene—biofilm of microorganisms” system; (**C**)—the formation of working electrodes based on a suspension of microorganisms and in the form of a biofilm and generation signal.

At the first stages, oxygen electrodes were used to create BOD biosensors [116,117]. The presence of luminescent genes in some bacteria, which leads to luminescence in the presence of various toxic compounds, made it possible to use optical signal converters to determine BOD. For example, luminescent detectors have been used to create BOD sensors based on the bacterium *Photobacterium phosphoreum* IFO 13896 [120] and luminescent recombinants of *E. coli* [121]. Luminescent bacteria, as a rule, do not have a wide substrate specificity due to the peculiarities of their own metabolic pathways, so they have not been widely used to determine BOD [122]. Since dissolved oxygen in the sample can distort the readings of the oxygen sensor during the measurement of BOD, mediator biosensors based on the use of special redox compounds capable of transferring electrons from the bioreceptor to the electrode as a result of oxidation–reduction have become more widespread [123,124]. As described in article [125], the typical response generation process in an amperometric sensor using a ferrocene mediator is presented (Figure 13). The basis of such electrodes is most often made up of various variants of carbon matrices [126,127].

The formation of biofilms on the electrode makes it possible to ensure the stability of the microbial community of the bioreceptor. For example, [128] presents a biofilm reactor that was fabricated via a cultivation process using naturally occurring microbial seeds from on-site surface water. The principle of determination was based on the fact that the oxygen concentration in the test sample was considered as a reference, and the oxygen consumption of the biofilm reactor (BFR) was calculated from the difference between the reference and the sample of effluents from the BFR. The system could operate continuously and stably for at least 30 days without human intervention. The biofilm reactor is a promising development, as it requires minimal maintenance and is easy to operate. And the cultivation of microorganisms on the electrode in solutions with increasing salinity makes it possible to monitor BOD in both fresh and salt water [129].

Electron transfer in associations of microorganisms can be difficult and unstable. Therefore, the creation of biofilms that ensure effective charge transfer in the system is an urgent task for researchers. The solution to this situation was the use of microorganisms capable of creating EABs. A group of metal-reducing bacteria of the genus *Geobacter* is capable of rapid and efficient direct electron transfer through terminal cytochromes to the electrode [130]. By varying the source of the nutrient substrate, biofilms with the necessary microorganisms can be formed; for example, the dominance of *Geobacter* in mixed cultures and such a biosensor can be used to determine BOD in the range from 174 mg/L to 1200 mg/L [131]. The detection of the ability of *Shewanella loihica* PV-4 cells to carry out bi-directional extracellular electron transfer led to the creation of a biosensor for simultaneous detection of BOD in the range from 0 mg/L to 435 mg/L and nitrates in the range from 0 mg/L to 7 mg/L [132].

Modification of biofilms with nanomaterials makes it possible to create electroactive biofilms based on microorganisms that are not capable of independent electron transfer to the electrode. For example, in [39] microorganisms of activated sludge grown on the surface of a graphite-paste electrode were modified with carbon nanotubes. The lower limit of detection for the presented biosensor based on an electroactive biofilm of activated sludge was 0.41 mg O_2_/dm^3^, which makes it possible to analyze almost any water sample, including surface water samples.

Modifying the biosensor design can improve the BOD detection systems. Thus, in [133], a self-adaptive system for determining a BOD bioreaction is presented, consisting of an “intestinal-like” microfluidic spiral bioreactor with a self-renewing biofilm. Due to the spontaneous surface adhesion of microorganisms from the environment, the biofilm was colonized in situ on the inner surface of the microfluidic spiral bioreactor. Using environmental domestication during each measurement of a real sample, the biofilm was capable of self-renewal to adapt to environmental changes.

**Figure 14:**
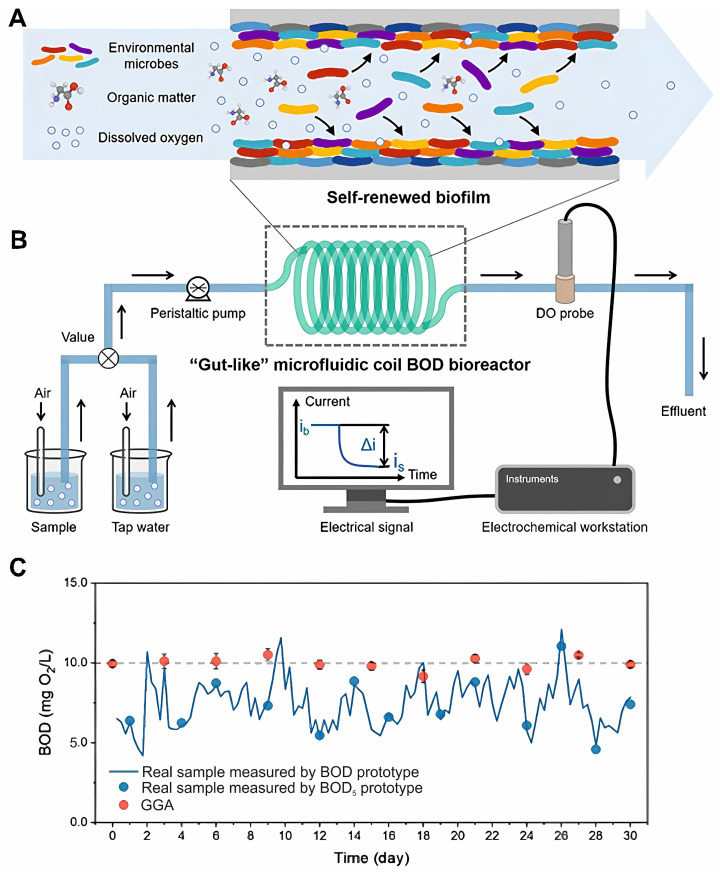
(**A**) Schematic of the in situ cultivation and environmental domestication of the self-renewed biofilm. (**B**) Diagram of the BOD bioreaction sensing system consisting of a “gut-like” microfluidic coil BOD bioreactor and a dissolved oxygen (DO) probe to monitor the DO consumption in the effluent for rapid BOD determination. (**C**) Long-term monitoring performance of real samples with accuracy evaluations compared by the standard BOD_5_ method and practical stability of the BOD prototype tracked with 10.0 mg O_2_/L GGA standards. The error bars represent the deviations of three successive measurements for the proposed method. Reprinted with permission from ref. [133] © 2023 Elsevier B.V. All rights reserved.

Figure 14 presents the schematic of the in situ cultivation and environmental domestication of the self-remixed biofilm and shows the possibility of long-term monitoring of BOD. Authors reconfigured the BOD bioreaction sensing system by arranging independent bioreactor and transducer units. This novel split design enables tailoring a suitable environment for biofilm formation, and the biodegradation efficiency can be easily adjusted by optimizing the sample injection rate and the inner diameter of the biomimetic gut.

Most of the biosensors currently presented in the literature for determining BOD are combined with biofuel cells, performing several functions simultaneously—BOD quantitative analysis, wastewater treatment, and electricity generation. Table 1 shows the MFC-based biosensor prototypes for determining the BOD index. In the biosensor models presented in the table, a biofilm is formed on the anode. As a rule, these are consortia of microorganisms isolated from activated sludge or wastewater. The organic substances in the sample serve as a carbon source for them. If nitrates are present in the samples, the sensor may give a false BOD value. To get rid of this problem, many researchers form a biofilm not only on the anode, but also on the cathode. For example, in [134], the biofilm was formed on the anode, where the dominant types of bacteria (more than 92% of the total population) were *Bacteroidetes*, *Proteobacteria*, and *Firmicutes*, and on the cathode, where *Bacteroidetes*, *Proteobacteria* and *Actinobacteria* prevailed (more than 84% of the total population). The main nitrogen removal processes in MFC biosensors involved heterotrophic denitrification and ammonification at the anode, ammonium transport to the cathode chamber, and bioelectrochemical autotrophic denitrification at the cathode. Using mathematical and graphical signal processing methods, it was possible to establish more accurate BOD values of samples in the range of 20–500 mg/L.

Inoculation of the anode with electrogenic and nitrite-reducing bacteria made it possible to create an electrochemical biosensor of dual function [148]. Simultaneous determination of BOD concentrations (in the range of 5~100 BOD L^−1^) and nitrites (in the range of 0.05~16 mg NO_2_^−^-N L^−1^) for 20 min and maintaining stable performance over 200 tests is of great importance for adjusting wastewater treatment strategies in real time.

Other researchers, for example Zhufan Lin and co-authors [149], introduced a new parameter—bioelectrochemical oxygen demand (BEOD), defined as the amount of oxygen required to degrade organic pollutants in this bioelectrochemical process. BEOD and BOD are related but not equivalent, and they characterize the bioelectrochemical degradability and biological degradability of organic matter, respectively. Therefore, in order for the EBS to accurately measure the BOD of wastewater, the bioelectrochemical degradability of organic pollutants needed to be considered when correlating electrical signals with BOD. As a result, the biosensor could measure BOD in the range of 4–160 mg/L with a measurement error of less than 4.4%, i.e., maintaining and accounting for an additional parameter increased the accuracy of the analysis. At the same time, this is the first biosensor that has successfully detected BOD in real wastewater with non-fixed organic components and has demonstrated detection accuracy in real wastewater < 14.6%.

Another way to improve the accuracy of the analysis is the use of various programming methods for processing data obtained from biosensors, for example, neural networks and time series analysis [150]. The authors use mathematical methods to analyze the biosensor response profile, taking into account the rate of signal change, and the area and height of the peak.

As can be seen from Table 1, the upper limit for determining BOD often does not exceed 400 mg/L, which is associated with saturation of the biofilm with the substrate. One solution to increase the detection range may be to use an array of several MFCs linked hydraulically in series, for example, as in [151]. This multi-stage configuration of the MFC made it possible to expand the detection range to 720 mg/L BOD_5_ (1175 mg L^−1^ COD). Thus, the biosensor can determine BOD concentrations ranging from those typical of municipal wastewaters up to those found in certain industrial wastewaters. Using a periodic and multi-stage flow mode, the authors managed to achieve biosensor operation for more than 800 days [152].

### 5.2. Biosensors for the Determination of Heavy Metal Ions

Heavy metals such as Hg, Pb, and Cd are extremely dangerous for humans and animals, because they can cause pronounced symptoms of poisoning even in low concentrations of the order of 1.0–10 mg/L, and also have the ability to accumulate in living organisms [153]. Heavy metals can alter physiological processes in cells [154], block their metabolism [155], and some of them have a carcinogenic effect [156]. The metabolism of bacteria in biofilms is usually also suppressed by the presence of heavy metals, which can be detected using optical or electrochemical transducers. Biofilm-based biosensors allow for cheap, simple, and reproducible measurements that can provide rapid screening of heavy metals and their toxic effects in aquatic environments. In particular, a biosensor based on electrochemically active *Rhizobium*-MAP7 and *Rhodotorula* ALT72 biofilms was presented in [157], which was used simultaneously to detect Cr6+ and Cd2+ ions in aqueous media and to remove them. Cyclic voltammetry was used as a registration method, and a decrease in the activity of the biofilm contained on the surface of the electrodes was estimated, depending on the presence of metal ions in the microbial culture. Electrochemical biosensors for the determination of heavy metals are also presented based on biofilms of *Geobacter* [158], *Pseudomonas* [159], as well as mixed communities from anaerobic sludge [160,161].

**Figure 15:**
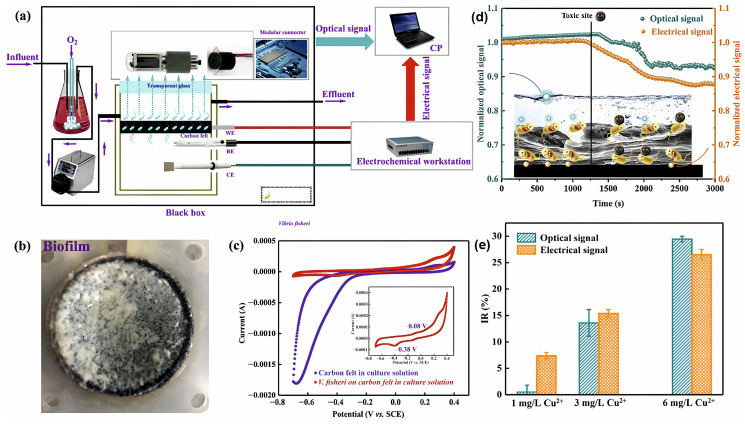
(**a**) Schematic diagram of dual-signal biosensor based on *Vibrio fischeri* biofilms for Cu(II) ion detection. (**b**) *Vibrio fischeri* biofilm, (**c**) cyclic voltammograms of biofilm, (**d**) reduction in the signal level when exposed to Cu(II), and (**e**) inhibition ratio of optical and electrochemical biofilm response. Adapted from ref. [163].

Not only electrochemical, but also optical sensors are used to determine the content of heavy metals. So, in [162] a Pulse Amplitude-Modulated, fluorometry–based biosensor was developed, used to quickly assess the possible acute and chronic effects of heavy metals in river biofilms. Qi et al. [163] used a biofilm of luminescent bacteria *Vibrio fischeri* to provide a simultaneous electrochemical and optical signal for the toxic effects of Cu(II) (Figure 15). At the same time, the optical detection method turned out to be more sensitive than the electrochemical one. However, the developed biosensor proved to be unstable when exposed to high concentrations of toxicants; concentrations of Cu(II) above 6 mg/L led to irreversible changes in the biofilm and permanent damage to the biosensor.

### 5.3. Biosensors for the Determination of Pesticides

Pesticides are widely used for pest control around the world, especially in agricultural developing countries. It is known that many of these pesticides are toxic and at the same time have high solubility, which leads to extensive contamination of groundwater and wastewater with these substances [164]. The implementation of an early warning system for the detection and monitoring of pesticides in drinking water and wastewater is essential to protect humans from potentially harmful effects. Microbial biosensors can provide fast and accurate inside testing for the presence of pesticides, and this field has been actively developing in recent years. The first studies on the negative effects of pesticides on the power characteristics of MFC biofilms were carried out more than 10 years ago; for example, the effect of the herbicide bentazone on the polarization curves of MFCs based on mixed-culture biofilms was studied [165]. A little later in [166], the possibility of using a miniature MFC based on a biofilm of activated sludge for the detection of atrazine to reduce the level of current generated by bacteria in its presence was studied. The MFC biosensor demonstrated a fast response to atrazine, with a sensitivity of 1.39 ± 0.26 ppm^−1^ cm^−2^ and a lower detection limit of 0.05 ppm. An important point in the creation of biosensors for the detection of pesticides is the choice of microorganisms for biofilms. In 2023, Aiyer et al. [167] evaluated the possibility of using an MFC based on «weak electricigens» for real-time water quality monitoring. Various pesticides were used as model substrates, and the developed electrochemical sensor was responsive within minutes at all concentrations tested (0.05–2 ppm). Native electroactive microorganisms, which are found in excess in domestic wastewater, were used in the research (including members of *Enterobacter*, *Pseudomonas*, *Klebsiella*, and *Delftia*). Thus, this study offers a new and promising approach to the application of microorganisms that are not commonly used in MFCs due to the low electrical power generated by them.

**Figure 16:**
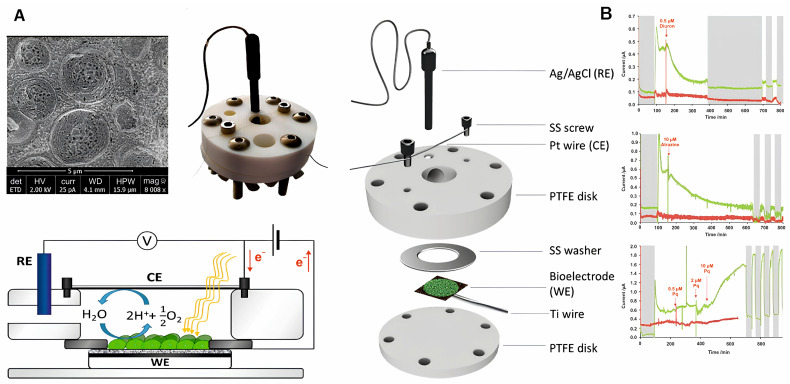
Setup used for photo-bioelectrochemical detection of pesticides (**A**). Effect of diuron, atrazine, and paraquat on photocurrent (**B**). The periods of darkness are represented with grey backgrounds. The biosensor was clamped together, with a stainless-steel (SS) washer. The bioelectrode (working electrode, WE) was clamped using two PTFE disks which also held the platinum wire (counter electrode, CE) and the Ag/AgCl reference electrode (RE). The stainless-steel washer ensured electrical connection between the bioelectrode and the titanium wire. Reproduced with permission from ref. [168] © 2019 by the authors MDPI.

Photo-bioelectrochemical sensors for detecting pesticides are built according to a similar principle. Currently, they are not as common as electrochemical ones, but nevertheless, research is being conducted in this direction. For example, the possibility of detecting three common herbicides (atrazine, diuron, and paraquat) to reduce the level of photocurrent of biofilms of the cyanobacterium *Synechocystis PCC6803 wt*. was studied [168]. It is worth noting that the detection mechanism was different for different herbicides; if atrazine and diuron inhibited the biosensor signal, then paraquat temporarily increased its signal by almost two times, because it was able to play the role of a redox mediator (Figure 16). Nevertheless, this device provided reliable detection of herbicides at the micromolar level, which are concentrations relevant to environmental analysis.

### 5.4. Biosensors for the Determination of Antibiotics

Antibiotics used to treat humans, plants and animals are only partially metabolized in their bodies and, therefore, can enter the environment in various ways. Residual amounts of antibiotics in the environment can lead to negative consequences, disrupting the growth and balance of aquatic ecosystems, increasing the resistance of pathogenic bacteria, as well as re-entering the human body with drinking water. Therefore, there is a need to develop devices that can quickly and effectively identify many different representatives of antibiotics.

The principle of using microbial biosensors to detect antibiotics is also based on their inhibitory effect. Thus, in [169] it was proposed to use a small microfluidic device to assess the content of various toxic substances in wastewater. The device has been tested on various toxic reagents (sodium cyanide, imidazole, and sodium azide), and it has been shown that the sensor can detect imidazole at the range of 0.02–0.4 mM. The work of Wu et al. in 2014 [170] demonstrated the limitations of the applicability of MFC-based biosensors for the detection of antibiotics. They used a single-chamber MFC with an electroactive mixed-culture biofilm with the addition of various concentrations of tobramycin. At the same time, it was shown that when using antibiotic concentrations below 2 mM and above 6 mM, the biofilm did not react to its presence in any way, giving a response only in a fairly narrow range of concentrations. Another single-chamber MFC was used [171] for the detection of levofloxacin. Acetate was used as a substrate for the MFC, and the decrease in the current level of the MFC directly depended on the concentration of the antibiotic in the range of 0.1–100 µg/L. At the same time, the biofilm on the anode demonstrated impressive stability, ensuring the operation of the device for 14 months.

The main problem with the use of biofilms for the detection of antibiotics is that while antibiotics effectively inhibit the growth of bacteria in the aquatic system, they can also affect biodiversity in it. They can not only inhibit the biosensor signal, but also change the composition of the biofilm if some microorganisms in its composition turn out to be more resistant to this particular substance. In this case, it is impossible to predict how the sensor will behave when exposed to a new specific antibiotic, and its repeated use is also difficult, because the parameters of the biosensor will almost certainly change due to changes in the composition of the biofilm after each use.

Comparison of the main parameters of some microbial biosensors for the determination of antibiotics, pesticides, and heavy metal ions is presented in Table 2.

### 5.5. Biosensors for the Determination of other Pollutants

There are many other substances that can pollute reservoirs and pose a threat to humans or animals. Biofilm-based biosensors can be used as a primary tool for assessing the overall quality of water or for finding specific pollutants (cyanides, azides, polychlorinated biphenyls, polycyclic aromatic compounds, etc.) [189,190]. However, they must necessarily be combined with more accurate and selective analysis methods, because for the most part, they are only able to provide an early warning about the deterioration of the quality of a particular water sample, but are not able to provide accurate information about the content of dangerous substances in it. In particular, in 2015, a µL-scale microbial fuel cell-based biosensor for water quality testing was developed [191]. A *Shewanella oneidensis* film was used as a biocatalyst, and formaldehyde was used as a model toxicant. Rapid current responses were detected over a concentration range from 0.001% to 0.1%, but at formaldehyde concentrations above 0.1%, the effect of the toxicant on the biofilm became irreversible, as a result of which the biosensor no longer restored its activity. A similar biosensor for the determination of formaldehyde was introduced two years later [192], but in the format of a screen-printed electrode. The sensor was able to detect 0.1% formaldehyde content; however, it had the same problems: low selectivity and reproducibility. A variant using the MFC biocathode as a sensor electrode for formaldehyde detection was also proposed and it was shown that its sensitivity is higher (7.4 ± 2.0 to 67.5 ± 4.0 mA(%^−1^)cm^−2^), than the sensitivity of a similar bioanode (3.4 ± 1.5 to 5.5 ± 0.7 mA(%^−1^)cm^−2^) [193]. There are also biosensors based on artificially formed biofilms. In [107], bacteria *S. oneidensis* were mixed with magnetic Fe_3_O_4_ nanoparticles to create a magnetically constructed EAB biofilm. The developed biosensor proved to be effective both for the detection of phenol and for the determination of heavy metal ions in wastewater samples with a detection limit of 0.07 mg/L. A similar non-selective biosensor was proposed in [194], where three-day biofilms of *Pseudomonas aeruginosa*, *Staphylococcus sciuri*, and *Bacillus amyloliquifaciens* were used to detect phenol, catechol, and 1,2-dihydroxynaphthalene.

It should be noted that biosensors that do not use an inhibitory effect to detect organic pollutants are extremely rare, but still occur. They are advantageous compared to other devices in that they have much higher selectivity. In particular, a biosensor based on *Pseudomonas monteilii* LZU-3 was able to detect the content of 4-nitrophenol in industrial wastewater, since these microorganisms could use nitrophenol as a sole substrate [195]. The authors tested the selectivity of the sensor by adding other toxic compounds to wastewater—2-nitrophenol, phenol, toluene and zinc chloride—and showed that even toxicants similar in structure to 4-nitrophenol did not affect the sensor signal in any way.

### 5.6. Biosensors for the Determination of Volatile Fatty Acids (VFAs)

Biofilm-based biosensors can be used to identify not only toxic substances, but also to monitor important indicators of biotechnological processes. For example, controlling the concentration of volatile fatty acids is one of the most important aspects in monitoring anaerobic fermentation processes. Usually, detection of intermediates of metabolic fermentation pathways—acetate, butyrate and propionate—is used for such monitoring [196]. Traditional methods of VFA detection are different types of chromatography, which means there is a need to develop fast and cheap methods of rapid analysis. In 2013, it was shown [197] that there is a correlation between the concentration of VFAs in the medium and the level of power generated by MFCs. Accordingly, MFC-based electrochemical biosensors can be used to determine the concentration of various types of VFAs. Jin et al. [198] in 2017 created a sensor that made it possible to measure the concentration of a VFA (acetate) in a wide range of 400–8200 mg/L. In addition, the sensor had high selectivity, because complex organic substances were delayed by the anion exchange membrane, which passed only VFA. There are also sensors that determine the amount of VFAs, rather than their individual concentrations. Thus, in [199] it was proposed to use a complex biofilm based on the bacteria *Geobacter*, *Hydrogenophaga*, *Pelobacter*, *Chryseobacterium*, *Oryzomicrobium*, and *Dysgonomonas* to detect the total content of VFAs in samples. It was shown that the sensor had a linear response to concentrations from 3 to 14 mM, and the measurement time was from 2 to 5 h.

Thus, the list of chemical compounds that can be determined by biofilm biosensors is quite wide, while the selectivity of individual models remains questionable. As we mentioned earlier, the formation and properties of biofilms depend on the environmental conditions and on the substances contained in them during growth. Therefore, by adding different compounds at certain stages of biofilm development, it is possible to regulate the sensitivity and selectivity of microorganisms in the biofilm, which may allow the creation of effective biosensors for specific tasks. However, since a biofilm is a complex biological system, it is quite difficult to fine-tune the biofilm and reproduce its properties repeatedly. This problem is still not completely solved and remains the most important topic for researchers of microbial biosensors. The “artificial” biofilms are getting more attention, because their parameters can be more strictly controlled in the process of sensor development. One of the methods that can be applied in the future to improve the selectivity of microbial biosensors and expand their scope is machine learning. With the help of computer analysis of bioelectric signals, it is theoretically possible to quantify several toxicants at once by finding the relationship between the types of current signals and information about toxicants. Currently, the first steps in this direction are already being taken; in [200], models were trained on biosensor signals that were exposed to mixed toxicants (MnCl_2_, NaNO_2_, and tetracycline hydrochloride (TCH)). The authors believe that thanks to the integration of machine learning, a microbial electrochemical sensor may be able to quantify several toxicants simultaneously, which will be the basis for a multiparametric determination of biotoxicity for environmental monitoring.

Genetically modified microorganisms, as mentioned above, open up new possibilities in the creation of biosensors. The removal or addition of certain DNA sequences to their genome can affect the efficiency of the biofilm formation process [201], the production of pili and exopolysaccharides [202,203], the conductivity of biofilms, which is important for electrochemical devices [204], as well as the fluorescence of biofilms, which makes it possible to use optical signal transducers of biosensors [205]. In addition, it is possible to block undesirable or induce desirable metabolic pathways in cells [206,207], which can change the substrate specificity of the biosensor in the direction necessary for researchers.

The great difficulty in forming a biofilm on the surface of the biosensor working electrode is the inability to strictly control its thickness and dimensions. One of the technologies that can help solve this problem is 3D printing [208]. Three-dimensional printing of microbes has recently become a new direction in biomedicine, and with it, new opportunities for the cultivation and manipulation of microbes have appeared [209]. The use of various 3D printing techniques allows you to adjust the ratio of volume to area of biofilms, biocatalytic activity of cells, as well as alter and augment transport of various components within a structure [210].

## 6. MFC Based on Microbial Biofilms

**Figure 17:**
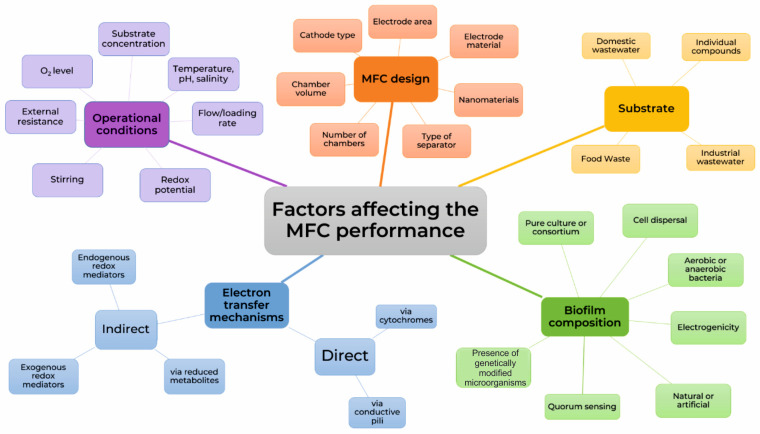
The main factors influencing MFC performance.

The wide substrate specificity of most microorganisms forming biofilms is a disadvantage for most biosensors based on them. At the same time, when using them as part of biofuel cells, this becomes rather an advantage. The ability to recycle a wide range of substances allows you to use more enzyme systems of microorganisms, ensuring longer-term operation of the MFC and a higher level of electricity generation. In our opinion, five categories of factors that have the most significant influence on MFC performance can be identified (Figure 17).

The ability of MFCs to generate electricity and treat wastewater tends to be directly related to the biofilms used, substrates, design features of MFC chambers and types of electrodes, operating conditions of the device, as well as electronic transport mechanisms in the system. This chapter examines in detail the latest developments in these areas of research, as well as possible areas that will be brought to the attention of researchers in the next 10 years.

### 6.1. Selection of Microorganisms and Characteristics of Biofilms

The selection of microorganisms for use in MFC anode biofilms is one of the most important tasks. The efficiency of substrate oxidation by microorganisms and the rate of electron transfer to the electrode determine the efficiency of electricity generation. Modern MFCs use aerobic and anaerobic microorganisms, and biofilms from both pure cultures and consortia [211]. At the same time, the use of mixed bacteria looks more promising, because they can clean contaminated samples as efficiently as their pure culture counterparts, but at the same time provide a higher level of generated power due to the oxidation of various substrates [212]. However, there are quite complex interactions within mixed communities, which can be both cooperative and symbiotic, as well as competitive or antagonistic [213]. Currently, pure-culture-based MFCs are mainly used for laboratory studies of the mechanisms of metabolism, electron transfer, and biofilm formation of individual strains, while consortia are used for the most efficient generation of electricity. Complex biofilms can consist of two cultures, the synergistic effect of which is well studied, or of dozens of different strains with extremely complex interactions. The most popular mixed crop used in MFCs to this day is active sludge [214]. However, its composition is constantly changing and difficult to reproduce, which means that it is impossible to fully predict the ways of processing substrates and the effectiveness of electronic transfer in each specific case. Therefore, much attention is paid to those compositions for which it is realistic to predict and explain ways to improve electricity generation. For example, in [215] the defined coculture of *Pseudomonas aeruginosa* and *Enterobacter aerogenes* was presented and optimal conditions were proposed for a 12-fold increase in the generated current density due to the fact that *P. aeruginosa* microorganisms isolated an electron transport mediator, which was then used by both strains to transfer electrons to the electrode. Another mechanism for improving electronic transport is the use of electrogenic bacteria in combination with non-electrogenic bacteria, capable of decomposing complex substrates. Kumar et al. [216] used *Bacillus licheniformis* to degrade xylan, and Li et al. [217] used *Lactobacillus plantarum* to purify azo dye wastewater. In both cases, bacteria of the genus *Shewanella* were used as effective electrons, and their co-cultures helped to build a metabolic chain for processing complex pollutants.

The other most promising areas of biofilm research to improve the efficiency of MFCs include the use of genetically modified microorganisms, the study of the quorum sensing effect in biofilms, as well as the creation of artificial conductive biofilms. As already mentioned in the section on biosensors, modification of the DNA of microorganisms opens up almost unlimited possibilities for controlling the parameters of biofilms—from changing its conductivity and the effectiveness of electronic transfer to introducing new elements into bacterial cells and blocking undesirable metabolic pathways [202]. This makes it possible to discover completely new substrates for generating electricity using MFCs. Thus, in [218], a highly efficient methane-powered MFC was proposed, which became possible thanks to a synthetic consortium that allowed converting methane directly into a significant electrical current. Global regulator engineering has been used for simultaneously optimizing multiple phenotypes (such as electroactivity and stress tolerance) of *Pseudomonas aeruginosa* P3-A-11 cells for use as part of MFCs [102]. This approach may allow the creation of more efficient electrogens, which in turn will expand the practical application areas of MFCs. Another key aspect of “synthetic biology” is the creation of artificial biofilms. Currently, artificial biofilms include systems in which bacteria are tightly immobilized into hydrogels and polymer matrices on various surfaces. But in fact, these systems cannot be called full-fledged biofilms, because they severely limit the growth of bacteria [219]. Nevertheless, the use of synthetic polymer matrices instead of exopolysaccharides secreted by bacteria often improves the electronic conductivity of the system and facilitates the access of substrates to microbial cells, which positively affects the power of MFCs based on such artificial biofilms. The main goal of researchers in the coming years should be to create new types of artificial biofilms in which a highly conductive polymer will provide a high rate of electron transfer, but at the same time not slow down the growth of bacteria, and perhaps even accelerate it. Such work is already underway; for example, Zhang et al. [220] used conductive polymer PMNT (poly(3-(3′-N,N,N-triethyloamino-1′-propyloxy)-4methyl2,5-thiophene hydrochloride) as an additive to the biofilm of *S. oneidensis*. This polymer not only improved extracellular electron transport, but also prolonged the life cycle of the bacteria, allowing the creation of a stronger and thicker biofilm on the surface of the MFC electrode. Further development of this area will make it possible to create more efficient biofilms with specified and easily reproducible properties, which will be of high importance for expanding the field of practical application of MFCs.

Finally, another interesting area of research is the study of the effect of quorum sensing in biofilms and its use in MFCs. Quorum sensing is a method of communication between microorganisms that can enhance the performance of MFCs by regulating electrode biofilms and coordinating their activities. The principle of its operation is based on the fact that bacteria secrete special signaling molecules that regulate gene expression, which leads to a change in the properties of the biofilm in response to various environmental factors. It has been shown that these molecules in the composition of MFCs strongly affect the ability of biofilms to self-assemble on the surface of the working electrodes of MFCs [221] and the ability to transfer electrons to mixed communities [222]. However, studies of the mechanisms of quorum sensing in the regulation of electrode biofilms are still at an early stage and many more tests need to be carried out in order to understand how this effect can be used to create biofilms with specified characteristics.

### 6.2. MFC Substrates

Substrates serve as food for bacteria in MFCs and therefore also play an important role in the development of MFC biofilms and in the performance of the device itself [223]. Substrates for MFCs are mainly divided into simple and complex ones. In the last decade, many simple substrates and wastewater types have been tested for energy production using MFCs. The MFCs, intended for practical use, are mainly aimed at processing complex substrates, especially various wastes—domestic [224], industrial [225,226], food [227], animal [228], agricultural [229], or pharmaceutical [230]. These substrates contain a mixture of various chemical compounds, and sometimes their own microbial communities. If we consider simple substrates, then the most popular of them are acetate, glucose, and lactate, as well as synthetic wastewater. Synthetic wastewater makes it possible to evaluate the effectiveness of the new MFCs in relatively realistic, but at the same time easily reproducible, compositions. Synthetic wastewater is widely used in scientific research due to the simplicity of varying substrate parameters such as electrical conductivity, loading rate, chemical composition, and pH [223]. Acetate is a highly effective substrate used by electroactive microbes as the primary carbon source. Moreover, in many metabolic pathways, acetate is the main end product. If one views the sole purpose of MFC development as maximizing power generation, then acetate will be the most effective substrate for efficient energy conversion in most cases [231,232]. Glucose is often used as an alternative carbon source in MFCs generating bioenergy. Its processing efficiency is much lower than that of acetate (3% conversion vs. 42%) [231]; therefore, it is most often used only as a model substrate for the transition from laboratory samples of MFCs to samples using synthetic wastewater or food wastewaters. The use of lactate as an energy source for MFCs is usually limited to wearable devices that use sweat as a power source for wearable biosensors [233]. In addition, there are works in which lactate or glucose are used as additional electrode donors for sulfate reduction using MFCs [234]. In general, we can say that MFCs as a tool for treating various wastewaters seems promising when expanding their scale, while simple substrates for MFCs are used only for laboratory testing, since even the most effective of them (acetate) does not allow MFCs to achieve efficiency comparable to traditional energy sources. The main task of researchers in such conditions is to increase the efficiency of wastewater treatment to above 95%, so that MFC technology can be used without expensive stages of secondary waste treatment before returning them to the environment.

### 6.3. Electron Transfer in MFCs Based on Biofilms

**Figure 18:**
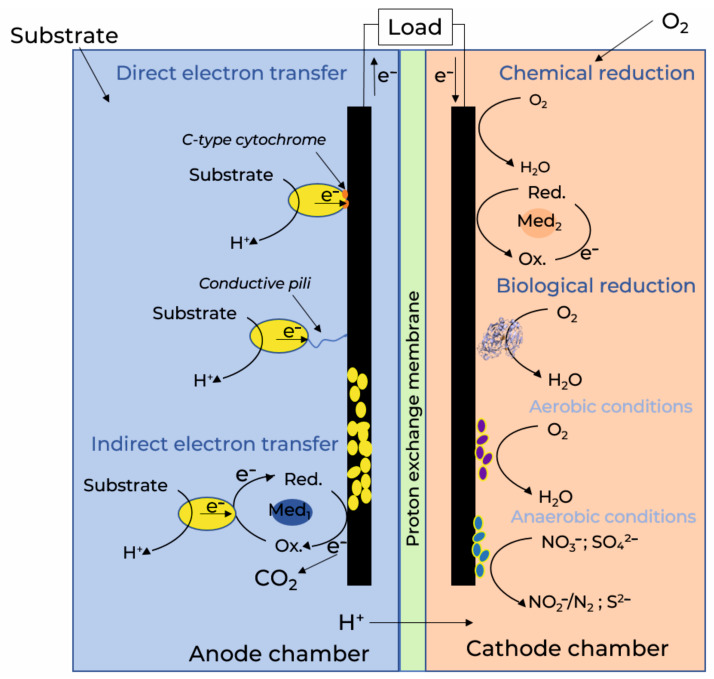
Mechanisms of electron transfer in a conventional two-chamber MFC.

An important factor influencing energy generation in MFCs is the method of electron transfer from bacteria to the electrode [202]. As is known, there are two types of electron transfer: direct and indirect. Direct transfer is carried out (1) with the help of redox proteins that are located on the surface of bacterial cells; (2) through conductive pili. Figure 18 shows a schematic of a conventional MFC with different possible electron transfer pathways in the anode and cathode compartments. Biofilms in the cathode compartment can operate under aerobic or anaerobic conditions, but enzyme-based cathodes or non-biological cathodes using electron transport mediators and air cathodes are also common.

If direct electron transfer is carried out, it is necessary to have a highly active microbial consortium on the anode, or a pure culture capable of such electron transfer. In addition, it is necessary to ensure close contact with the surface of the electrode. The density of the biofilm will also be an important factor, since in fact the first layer of bacteria adjacent to the electrode will be the most electroactive. The ability of bacteria to form pellets allows the transfer of electrons in the biofilm from microorganisms more distant from the electrode surface.

Mediated transfer uses mediators. They can be both secondary metabolites and mediators secreted by microorganisms during metabolism, or redox compounds specially added to MFCs that are non-toxic to microbes.

In MFCs, such electroactive bacteria as representatives of the genera *Shewanella* and *Geobacter*, capable of direct transfer, have been mainly used to create biofilms [58]. Either consortia specially enriched with these cultures have been used, or conditions (variations of the electrode material and biofilm growth conditions) have been created for the predominance of desirable microorganisms in the film [235]. Modern research has led to the discovery of the ability for direct transfer in representatives of other genera, which significantly expands the scope of MFC applications based on them [236]. Electroactivity has been confirmed for representatives of the genera *Betaproteobacterium*, *Chloroflexi*, hyperthermophilic *Archaea*, and also in iron oxidizers like *Acidithiobacillus ferooxidans*, nitrate reducers like *Pseudomonas alkaliphila*, sulfate reducers like *Desulfobulbus propionicus*, acetogens like *Sporomusa* ovata, methanogens like *Methanosarcina barkeri*, and photoautotrophs like *Rhodopseudomonas* palustris or *Prosthechocloris aestuari* [237]. Currently, to enhance the electrical conductivity of a biofilm, various conductive polymers can be introduced into it during growth [238], and also nanomaterials [41]. The use of three-dimensional nanostructures makes it possible to significantly increase the contact area of bacteria and the electrode, which leads to an increase in the efficiency of extracellular electron transfer, and, accordingly, to an increase in current generation [239].

The use of genetic modification makes it possible to create hybrid strains capable of expanding the substrate specificity of the microorganism and the possibilities of its use in MFCs [240,241]. The study of the mechanism of extracellular electron transfer in the *Bacillus megaterium* strain (LLD-1) showed that flavins in the culture suspension of the LLD-1 strain are able to act as electron carriers, enhancing electron transfer from cells to the electrode [242]. Knowledge of the mechanism made it possible to increase energy generation by adding exogenous flavins. The presented model for improving the effectiveness of MFCs can be used for other representatives of Gram-positive bacteria.

The study of electron storage mechanisms also opens up broad prospects. Thus, in [243], data on studies related to sulfide-oxidizing bacteria are summarized. These bacteria can transfer electrons between the sulfide ion and the electrode. Bacteria, under both variable and constant redox conditions, removed sulfide from the solution in the absence of an electron acceptor, and when transferred to an electrochemical cell, electrons were released onto the anode. The electric current measured in the anode circuit was higher in bacteria that were subjected to variable redox conditions than in bacteria that were subjected to constant redox conditions. This clearly shows that these bacteria were able to store electrons, since the oxidation reaction (sulfide to elemental sulfur) was separated from the reduction reaction (electron transfer to the electrode as the final electron acceptor) both in time and in space. Thus, it is possible to influence the selectivity of sulfide to sulfur conversion, and knowledge of electron storage mechanisms can lead to the development of new MFC power management strategies.

### 6.4. Operational Conditions of MFCs

Many studies have been conducted to evaluate the effectiveness of MFCs when changing certain operating parameters. These parameters include the substrate concentration [244] and loading rate [224], the level of dissolved oxygen in MFCs [245], conductivity, pH, temperature and salinity of the anolyte [246], the presence of mixing [247], and external resistance [248]. For the most part, researchers vary one or more parameters when developing new MFCs, but a fairly small number of works are devoted to large-scale studies of the effect of one or another parameter on the general patterns of functioning of microbial biofilms. Most of the papers in the literature are devoted to the effects of pH on the formation and functioning of anode and cathode biofilms in the composition of MFCs, since this parameter is one of the most easily variable. In particular, the effect of pH on the stratification of the bacterial community in the MFC cathode biofilm under alkaline conditions was studied in [249]. The authors showed that, depending on the pH, the content of aerobic bacteria in the upper, middle, and lower layers of the biofilm varied in percentage, which made it possible to further use pH as one of the tools for regulating the microbial composition of biofilms. Varying the flow rate and culture time allowed changing the response of microorganisms in MFCs to toxic compounds [250]. This can be useful not only for creating MFCs that will be able to purify toxic wastewater, but also for MFC-based biosensors aimed at detecting toxic compounds.

The concentration of the substrate varies in almost any study on MFCs; however, it is interesting that the maximum concentration does not always correspond to the most effective operation of MFCs. Thus, in [251] with an influential substrate concentration of 0.81 g/L, COD removal efficiency, current density, and power density of an MFC were 84.6%, 162.6 mW/m^2^, and 468.7 mA/m^2^, respectively. At the same time, with a doubling of the substrate concentration, these parameters decreased to 62.8%, 74.8 mW/m^2^, and 183.58 mA/m^2^. The sharp decrease in energy production is explained by self-inhibition at higher carbon loading rates, since the microorganisms in the biofilm are not enough to work with an excess of substrate, and the accumulated load affects the efficiency of anaerobic microorganisms by increasing mass transfer losses. Similar results were obtained in [252], where with an increase in COD concentration from 2000 mg/L to 10,000 mg/L, the purification efficiency decreased from 84 to 79%.

Temperature is also a very important factor in biotechnological processes of energy generation by microorganisms. Temperature affects the kinetics and thermodynamics of biochemical reactions in biofilms, as well as the rate of biofilm formation, which ultimately affects the overall performance of MFCs [253]. There is a consensus that an increase in temperature in most cases has a positive effect on the output power of MFCs, since temperature accelerates the metabolism of microbes [254,255]. Nevertheless, changes in the biofilm growth rate, its metabolism, as well as in the ohmic resistance of MFCs, depending on temperature, are not linear; therefore, the range of 30 to 45 °C is considered optimal [256]. There are exceptions to this rule, so Ren et al. it was shown that their mini-MFC based on a *Geobacter sulfurreducens*-dominated mixed inoculum demonstrated the highest efficiency in the temperature range 49–53 °C with a remarkable current density improvement of 282% compared to room temperature performance [211].

### 6.5. MFC Design

When developing MFCs, the design of the device is chosen first. This includes the number of chambers, their material and volume, the separation membrane, and the material and dimensions of the electrodes. Each of these factors directly affects the characteristics and efficiency of the device being developed. A significant number of reviews are devoted to various variants of MFC designs developed to date [257,258,259,260]; therefore, in this section of the review we will consider only those aspects of MFC design that are directly related to biofilms.

Biofilms in MFCs are formed at the anode or cathode, so the main factor influencing its growth and development is the choice of electrode material. Electrodes for MFCs must have biocompatibility, a large surface area, and low resistance. In most cases, MFC electrodes are made of various carbon materials [261], conductive polymers [262], nanomaterials [263], and composite materials [264]. To increase the active surface area of the biofilm, one option is to use porous structures and three-dimensional electrodes [265]. A special microclimate is created inside such electrodes, allowing bacteria to remain active longer, which has a positive effect on the productivity and duration of MFC operation [266].

When creating MFCs, researchers face a double task: to ensure the growth of biofilms on the anode and/or cathode, and at the same time eliminate biofouling of separator membranes. This phenomenon leads to serious consequences as it increases the ohmic resistance of the electrode and the resistance to charge transfer and prevents proton transfer, which leads to a rapid decrease in the energy characteristics of the system [267]. Various approaches are used to solve this problem: surface modification associated with the creation of antifouling coatings [268], the use of composite materials [269], and methods of chemical and physical purification using surfactants, acids, hydroxides, and ultrasound [270,271]. Modification of the electrode surface leads to an increase in MFC power by 6–7 times in the long term, whereas its regeneration strategies lead to the restoration of up to 100% of the initial characteristics. Further research includes approaches such as the design of MFC chambers based on hydrodynamics and plasma purification. Nevertheless, the biofouling process is still insufficiently studied in the field of bioelectrochemistry and requires systematic improvement [272].

Despite significant advances in the development of MFCs, their average output power is still not high enough for most practical applications. The main directions for improving the efficiency of the MFCs being developed are increasing the size of the device, stacking multiple cells in series or parallel, as well as using devices accumulating and storing energy generated by the MFC. Large-volume devices are presented in the literature (>1000 L [273,274,275]); however, it is not always a simple increase in the size of the electrodes and cells that leads to a proportional increase in the power of the device. At the same time, in large-volume MFCs, it is more difficult to control parameters such as the composition of the biofilm, contamination of the membrane and cathode, as well as the stability of measurement conditions.

When using the stacked MFC system, individual cells can be connected in series or in parallel. As a rule, a serial connection leads to an increase in the generated voltage, and a parallel connection leads to an increase in current [276]. There are known works in which serial and parallel connection of individual MFC cells is combined—up to 96 modules [277]. In fact, this approach is also based on increasing the total surface area of the electrodes and the volume of the reactors, but with this configuration it is much easier to track changes in MFC parameters during continuous operation. Stacked systems make it possible to achieve more complete wastewater treatment when using this as a substrate. Thus, [278] showed an increase in the COD removal rate to 90% compared to 60% for single modules, and in [279] when using a cascade system of three stacks of three cells each, the cleaning efficiency increases to 70–90% compared to 33% for a single MFC.

Another method associated with the possibility of increasing the amount of energy produced by MFCs is associated with the use of special converter devices. To increase the output voltage of the MFC, power management systems have been developed, including DC-DC converters. The first generation could raise the MFC voltage to 1.8–2.4 V, which was not enough for most devices. Power management systems of the second generation, based on boost converters, could charge capacitors more quickly and raise the voltage to 3.3 V [280]. In [281], an ultra-low-power energy harvester specially designed for MFCs is presented. The minimum required input power was only 2.1 µW, which made it possible to effectively use even the most low-power MFC. In article [282], the author presents a power management system called Low-Voltage Booster followed by a Rectifier (LVBR) circuit that was developed to increase the low MFC output voltage (VMFC) to a directly usable level for charging power banks (3.7 V for a rechargeable lithium-ion battery and 5.1–9.2 V for supercapacitors). In [283], such a system not only increased the output voltage from several MFCs to the required level, but also disconnected individual MFCs from the circuit when their voltage fell below a predetermined threshold.

Thus, the literature analysis given in this section shows that intensive research is being conducted to improve the stability and effectiveness and expand the scope of practical applications of MFCs. The principle of operation of MFCs has been known for more than 100 years, starting with the works of Michael Cresse Potter [284], and interest in them has steadily grown since the 80s of the last century [285]. Since the 1990s, microbial fuel cells have been considered as one of the most promising tools for the treatment of various wastewaters with simultaneous energy generation [286], which has led to the creation of devices that have been effectively used in practice for several years. Currently, the conditions for the formation and growth of biofilms, the mechanisms of electronic transfer from bacteria to the electrode, the effect of nanomaterials and polymer gels on the efficiency of MFC, etc. are well studied. Therefore, many processes occurring in MFCs are quite easily predicted, which allows researchers to pay more attention to exploring the possibility of applying new directions to create efficient devices. Thus, the use of artificial intelligence for the modeling and optimization of various processes occurring in microbial fuel cells is presented in the work [287]. Artificial neural networks make it possible to avoid the need for complex analysis of the biofilm metagenome, and are also useful when scaling devices to establish relationships between input parameters and output power when conducting optimization studies [288]. The use of advanced power management systems in addition to MFCs allows you not only to collect and store energy from them, but also to manage the processes occurring in stacked MFC systems, efficiently distribute the energy generated by them, as well as combine fuel cells of various classes in one device, not only MFCs [289].

## 7. Conclusions

The topic of publications over the past 10 years shows the interest of the scientific community in the creation and modification of biofilms for various purposes, including the creation of bioelectrochemical devices. Currently, the conditions for the formation and growth of biofilms on various materials, the mechanisms of electronic transfer from bacteria to the electrode, the effect of substrate modification, as well as the introduction of additional nanomaterials and polymer gels into the biofilm composition on the efficiency of biosensors and MFCs have already been well studied. In addition, strategies for genetic modeling of the composition and properties of biofilms are actively used. The most interesting and developing area is the modification of microorganisms using genetic engineering, which includes not only working with indirect and direct EET genes, but also adding the property of electroactivity to initially non-electrogenic microorganisms. In addition, genetic engineering methods make it possible to expand the substrate specificity of known electrogenic bacterial strains, which in turn expands the possibilities of using these microorganisms. In addition to improving electroactivity, researchers are changing the morphology and parameters of biofilm formation itself, enhancing the synthesis of the polysaccharide matrix and the interaction between microorganisms.

One of the latest trends is the search for new organisms capable of biofilm formation. In future studies on applying microbial biofilms to bioelectrochemical devices, lichens, which are natural microbial biofilms, may attract attention. Lichens were once thought to be the first organisms to invade the land (though that theory of evolution has now been disproved [290]). The reason for this is that because they are symbiotic organisms consisting of cyanobacteria, which are producers in the ecosystem, and fungi, which are decomposers, they can grow anywhere on land as long as they have nutrients and other minerals.

There are not many studies applying lichens to the biosensors [291]. The reason for this is that lichens grow slowly and are sensitive to environmental changes, making them difficult to handle. However, from a long-term perspective, no other organism is better suited to monitor the environment. If lichens can be stably rooted and electrically connected on the electronic device as an electroactive biofilm capable of electron transfer, the bioelectrochemical device can stably monitor the environment without changing shape, using only the initial minerals, a small amount of water, and atmospheric CO_2_. Then, by equipping with solar panels and transmitters, the bioelectrochemical device would be possible to create a semi-permanent and autonomous environmental monitoring system.

Another area of development in this field is the creation of “artificial” biofilms that surpass natural biofilms in their characteristics and are more “easily customizable”. The entire array of information collected by researchers to date allows us to predict the properties and mechanisms of processes occurring in biofilms and on electrodes. The use of artificial intelligence opens up opportunities for scaling, regulating, and controlling the processes occurring in bioelectrochemical devices, as well as including these devices in smart home systems, the Internet of Things, and so on.

In summary, great progress has been achieved in the study of biofilms and their application for the development of efficient analytical devices. In the field of biotechnological process control and environmental monitoring, microbial biosensors and biofilm-based MFCs have already found their niche and are able to compete with traditional analytical methods or complement them due to their unique properties. The next challenge for researchers should be the search for new applications for these devices. For example, great prospects open up when assessing the possibility of using such devices in medicine. Currently, enzyme and affinity biosensors are already widely used in clinical and sports medicine, but the use of microorganism-based devices is limited due to the fear of potential infection of the organism. At the same time, microorganisms and biofilms already present in the human microbiome can theoretically be used as biocatalysts. This will allow reducing the risk of infection and creating not only wearable analyzers, but also implanting various versions of bioelectrochemical devices directly into the human body.

## Figures and Tables

**Table 1:** Models of biosensors combined with MFCs to determine the BOD index.

The Composition of the Biofilm	The Time of Colonization	The Electrode	Detection Range	Analysis Time	Real Samples	Reference
*Shewanella loihica PV-4*	At least 5 days	Carbon cloth 2.5 cm × 2.5 cm	43.5–435 mg/L	1 h	Electrolytes containing various concentrations of sodium lactate	[132]
Microbial community with a predominant dominance of three species *Citrobacter freundii*, *Aeromonas hydrophila* and *Desulfovibrio desulfuricans*	From 22 to 140 h	Carbon brushes with a diameter of 2.50 cm and length of 2.50 cm	10–80 mg L^−1^	0.25 h	Wastewater	[135]
Microorganisms contained in contaminated water samples	2 months	A piece of not wet-proofed carbon paper 9 cm^2^	0–250 mg/L	0.67 h	Real contaminated groundwater	[136]
Bacterial inoculum	Secondary biofilm	Carbon cloth, coated with single-walled carbon nanotubes, the size of the anode was 10 mm × 10 mm	49–492 mg/L	-	Artificial wastewater	[137]
Effluent enriched with *Geobacter genus* (98%)	About 1 year	Carbon felt with a geometric surface area of 16 cm^2^	0–250 mg/L	2 min	Domestic wastewater from Waterloo wastewater treatment plant (ON, Canada)	[138]
Inoculated with mixed culture anaerobic sludge	4–6 weeks	Carbon felt 0.3 cm in diameter and 0.3 cm in thickness	Up to 300 mg L^−1^	10 min	Synthetic wastewater	[139]
Pre-cultured electroactive bacteria isolated from activated sludge	5 days	Carbon fiber veil with a total macro-surface area of 1250 cm^2^, wrapped around a ceramic cylinder	Up to 149.7 ± mg O_2_ L^−1^	5 min	Water samples from the Cotswold Water Park (UK)	[140]
Bacterial community with a dominant presence *Geobacter*	-	Carbon felt size 2 cm×2 cm	25–400 mg L^−1^	-	Synthetic wastewaters	[141]
Activated sludge with a predominant content of genera *Aquabacterium* and the aerobic denitrifier *Thauera*	2 weeks	A stainless-steel mesh size 220 mm × 760 mm × 0.5 mm	40 to 200 mg/L	6 h	Swine wastewater	[142]
A microbial community with a predominance of *Proteobacteria*, *Firmicutes*, and *Bacteroidetes*	48 h	The aerogel of carbonized *Luffa cylindrica* (LC) was used as the scaffold for loading biofilm and FeS_2_ nanoparticles (FeS_2_NPs) were employed to modify this aerogel (FeS_2_NPs/GelLC)	6–30 mg/L	30–100 min	Water samples from Lake Chagan	[143]
*Geobacter*-enriched mixed bacterial culture from anaerobic digester sludge	40–44 h	Carbon cloth size 10 mm × 4 mm	20−490 mg/L	1.1 min	Wastewater samples from municipal wastewater treatment plant	[144]
Bacterial inoculum was obtained from effluent of an acetate-fed microbial electrolysis cell (MEC) mother reactor that had mixed bacterial culture from anaerobic digester sludge.	24 h	Carbon cloth size 10 × 10 mm	Up to 400 mg/L	10 min	Wastewater samples from the Toronto wastewater treatment plant and the Burlington wastewater treatment plant	[145]
Yeast	2 weeks	Carbon felt with a projected active area of 7 cm^2^	0−10 mg/L	30 min	The lake, river, and tap water samples	[146]
*Bacteroidetes*, *Proteobacteria*, and Firmicutes were the major functional bacteria (92%)	1 year	Carbon cloth with diameter of 2.8 cm	20 to 500 mg/L	15 min	Synthetic wastewaters	[147]

**Table 2:** Examples of microbial biosensors for detection of heavy metals, pesticides, and antibiotics.

The Composition of the Biofilm	The Time of Colonization	Working Electrode	Limits of Detection	Analysis Time	Real Sample	References
**Heavy Metal Ions**						
Activated sludge	10 days		Mg: 0.01 mg∙L^−1^; Pb: 0.1 mg∙L^−1^;	1 h	Inlet of the wastewater treatment plant	[172]
Engineered HJFbgrTM *Bacillus subtilis* biofilm	6 days	Biochar	Pb, Cu: 0.1 μM; Hg^2+^: 0.01 μM;	12 h	Contaminated soil	[173]
Microbial community from sediment soil, mostly *Proteobacteria*	30 days	Carbon felt	Cd^2+^, Zn^2+^: 1 mg/L; Pb^2+^, Hg^2+^: 0.5 mg/L	30 min	Lake water samples	[174]
Mixed microbial culture from industrial wastewater	2 weeks	Graphite	5 mg/L for Cu^2+^, Cr^6+^, Zn^2+^, Ni^2+^	2 h	Industrial wastewater	[175]
*E. coli* BL21 engineered to express genes with P_zntA_ promoter, which could sense zinc	120 h	Carbon felt	Zn: 20 μM	15 h	Synthetic wastewater	[176]
Enriched microbial culture from secondary sedimentation tank sludge	50 h	Carbon felt	Cd: 0.1 mg L^−1^ Cr^6+^, Zn^2+^, Cd^2+^: 1 mg L^−1^	30 min	-	[177]
Inoculated aerobic sludge	35 h	Carbon felt	Cu^2+^: 1 mg/L	5 h	-	[178]
**Pesticides**						
Anaerobic sludge	7 days	Carbon cloth, 0.32 cm^2^	Atrazine, 0.05–0.3 ppm	24.4 ± 7.7 min	-	[166]
Algae *Chlamydomonas reinhardtii*	24 h	Screen-printed electrodes modified with carbon black nanoparticles	Atrazine, 0.1 and 50 μM; RSD of 1.1%; storage stability up to 3 weeks	15 min	River water	[179]
Cyanobacterium *Synechocystis* PCC6803 wt.	48 h	A filter paper sheet was covered with seven layers of single-walled carbon nanotube paint	Atrazine, diuron, and paraquat; 10.7, 0.5, 0.7 mM; stability >20 days	250 min		[168]
Artificial multi-species biofilm from *E. coli*, *S. cerevisiae*, and *B. subtilis*	24 h	Platinum disc covered with a piece of PVA–alginate microbial biofilm attached	3,5-dcp—1 mg/L; Ametryn, acephate, and thiram—5 mg/L	20 min	River water, wastewater from garbage-treatment plant, and landfill wastewater	[180,181]
Algae *Scenedesmus obliquus*	24 h	Acid-treated carbon graphite felt	Atrazine—0.5 mg/L	2 h	-	[182]
Mixed biofilm from *Proteobacteria*, *Bacilli*, *Deltaproteobacteria*, and *Betaproteobacteria*	48 h	Aerogel of carbonized *Luffa cylindrica* with FeS_2_ nanoparticles	3,5-dcp—10 mg/L	30 min	Water from Lake Chagan	[143]
*Geobacter*-dominated mixed biofilms	15 days	Carbon cloth treated by ammonia	Avermectin and ivermectin—1.0 mg/L	70 min	-	[183]
**Antibiotics**						
*Escherichia coli*/pMTLacZ		Filter paper strips (1 × 4 cm)	Tetracycline; detection limits of 5.23–17.1 μg/L for water and 5.21–35.3 μg/kg for the EDTA soil extracts; range of 75–10,000 μg/L in water and 75–7500 μg/L in soil extracts	90 min	Water; soil extracts	[184]
*Escherichia coli* SN0301	>20 h	Microplate, fluorescence	8 pg/mL of meropenem and 40 pg/mL of imipenem; 1–10 ng/mL for penicillins and cephalosporins			[185]
Anaerobic digestion sludge	7 days	Three-dimensional porous pristine carbon fiber	Neomycin—0.01 mg L^−1^	1 h	Domestic wastewater treatment plant	[186]
*Pseudomonas putida* TSh-18 biofilm	4 days	Microplate, fluorescence	Ampicillin—0.5 μg/mL	24 h	-	[187]
Recombinant plasmids transferred into *Escherichia coli* DH5α	12 h	Microplate, fluorescence	Tetracycline—30 μg/L	1 h	Lake water and tap water	[188]
*Geobacter*-dominated mixed biofilms	15 days	Carbon cloth treated by ammonia	Chlortetracycline—1.0 mg/L	70 min	-	[183]

## Data Availability

Data are contained within the article.

## Abbreviations

BOD	Biochemical oxygen demand
MFC	Microbial fuel cell
EPS	Extracellular polymeric substances
EET	Extracellular electron transfer
EAB	Electroactive biofilm
QS	Quorum sensing
CNT	Carbon nanotubes
SEM	Scanning electron microscopy
VFA	Volatile fatty acids
